# Global Trends and Hotspots in Research on the Health Risks of Organophosphate Flame Retardants: A Bibliometric and Visual Analysis

**DOI:** 10.3390/toxics12060391

**Published:** 2024-05-27

**Authors:** Zhiyuan Du, Yuanyuan Ruan, Jiabin Chen, Jian Fang, Shuo Xiao, Yewen Shi, Weiwei Zheng

**Affiliations:** 1 Key Laboratory of the Public Health Safety, Ministry of Education, Department of Environmental Health, School of Public Health, Fudan University, Shanghai 200032, China; duzhiyuan0422@163.com (Z.D.); 22301020036@m.fudan.edu.cn (J.C.); 22301020015@m.fudan.edu.cn (J.F.); 2 NHC Key Laboratory of Glycoconjugates Research, School of Basic Medical Sciences, Fudan University, Shanghai 200032, China; yuanyuanruan@fudan.edu.cn; 3 Department of Biochemistry and Molecular Biology, School of Basic Medical Sciences, Fudan University, Shanghai 200032, China; 4 Department of Pharmacology and Toxicology, Ernest Mario School of Pharmacy, Environmental and Occupational Health Sciences Institutes, Rutgers University, Piscataway, NJ 08854, USA; sx106@pharmacy.rutgers.edu; 5 Shanghai Municipal Center for Disease Control and Prevention, Shanghai 200336, China; 6 Center for Water and Health, School of Public Health, Fudan University, Shanghai 200032, China

**Keywords:** organophosphate flame retardants, health risks, research hotspots, future trends, bibliometrics

## Abstract

Background: Organophosphate flame retardants (OPFRs) are compounds with a wide range of industrial and commercial applications and are mainly used as flame retardants and plasticizers. The global consumption of OPFRs has risen rapidly in recent decades, and they have been widely detected in environmental media. Unfortunately, OPFRs have been associated with many adverse health outcomes. The issue of the health risks of OPFRs is attracting increasing attention. Therefore, there is a need to review the current state of research and trends in this field to help researchers and policymakers quickly understand the field, identify new research directions, and allocate appropriate resources for further development of the OPFR health risk research field. Methods: This study statistically analyzed 1162 relevant publications included in the Web of Science Core Collection from 2003–2023. The internal and external features of the literature, such as publication trends, countries, authors, journals, and keywords, were quantitatively analyzed and visually presented to identify the research hotspots, compositions, and paradigms of the field and to horizontally and vertically analyze the development trends and structural evolution of the field. Results: The development of the field can be divided into three stages, and the field entered a period of rapid development in 2016. China (649 papers) is the most prolific country, followed by the United States (188 papers). The authors STAPLETON HM and WANG Y have the highest combined impact. International collaboration between countries and researchers still needs to be strengthened. Science of The Total Environment is the most frequently published journal (162 papers), and Environmental Science and Technology is the most frequently cited journal (5285 citations). Endocrine disruption, developmental toxicity, and neurotoxicity are the health effects of greatest interest. Conclusions: Future research is expected to be multidisciplinary, and research hotspots may involve a comprehensive assessment of OPFR exposure in the population, exploration of the mechanisms of endocrine-disrupting effects and in vivo metabolic processes, and examination of the health effects of OPFR metabolites.

## 1. Introduction

Organophosphate flame retardants (OPFRs) are organic esters of phosphoric acid containing an alkyl chain or an aryl group and can be halogenated or nonhalogenated. OPFRs have a wide range of industrial and commercial uses, mainly as flame retardants and plasticizers [1,2], and are commonly used in products such as textiles, construction materials, food packaging, and electronic devices [3,4,5]. Since the gradual restriction of the global use of polybrominated diphenyl ethers (PBDEs) beginning in 2003, the production and use of OPFRs have been growing rapidly worldwide [6,7]. The global consumption of flame retardants exceeded 2.7 million tons in 2022, with phosphorus flame retardants accounting for 28% of that amount [8]. OPFRs are added to products as additives rather than via chemical bonding, which results in OPFRs being easily introduced into the surrounding environment (e.g., air, dust, water, and soil) through volatilization, dissolution, and abrasion [9,10,11]. In recent years, a growing number of studies have shown that OPFRs may cause adverse health effects such as carcinogenicity [12,13], endocrine disruption [14,15,16], neurotoxicity [17,18], and reproductive toxicity [19,20] by inhibiting enzyme activities, disrupting the immune system and hormonal homeostasis. The potential risks of OPFRs to the ecosystem and human health are attracting increasing attention.

Several review papers on OPFRs have been published in recent years [5,9,21,22], most of which focus on one or two aspects of detection, environmental fate, or toxic effects of OPFRs. No studies have been conducted to integrate and analyze the field of OPFR health risk research at the macro level using a database of relevant studies, making it difficult to arrive at a complete picture of OPFR health risk-related research. Bibliometric analysis is an effective means of integrating and analyzing big data, combining statistical and mathematical methods to analyze existing knowledge databases from a macro perspective and provide systematic, objective, and reproducible evidence-based insights for assessing the current status and trends of the research field [23]. Its use has been extended to diverse fields of research, including environmental and health research [24,25,26,27].

This work analyzed studies related to health risks of OPFRs during the last 20 years (2003–2023) based on bibliometrics, aiming to (1) present a full picture of the research field and promote communication and collaboration in specific fields by analyzing the country/region, institution, and authors of the publications [28]; (2) provide a multidimensional evaluation of authors and journals, which can help researchers quickly locate relevant research directions, fields, and professionals, helping researchers and institutions make informed decisions on collaboration, funding, etc. [29]; and (3) identify the main research directions in the field by analyzing keywords and highly cited studies, determine the current status and development trends of research in a specific direction, and promote evidence-based decision-making, helping researchers and policymakers identify emerging research directions and allocate resources accordingly [30].

## 2. Materials and Methods

### 2.1. Data Source

The Web of Science Core Collection (WOSCC) is a comprehensive database of high-quality journals in all disciplines and is the most commonly used database for scientific or bibliometrics analysis [31,32]. To ensure the comprehensiveness and reliability of the data sources, the search options for scientific data mining in this study were limited to the following: database = “Web of Science Core Collection” and editions = “Science Citation Index Extended” and “Social Sciences Citation Index”. The search terms for publications related to the topic of the health risks of OPFRs were as follows: (“organophosphate flame retardants” or “organophosphorus flame retardants” or “OPFRs” or “organophosphate esters” or “OPEs” or “organophosphate triesters”) AND (toxicity or health or risk). The time span was 2003–2023, the language was limited to English, and the type of document was an article or review article. The last search was made on 12 January 2024. The obtained documents were imported into Zotero (version 6.0) and manually checked; duplicates and studies with incomplete information were deleted, and documents that were irrelevant to the topic of the study were eliminated (more details can be seen in Figure S1). A total of 1162 documents were ultimately obtained. The full records and cited references of the included studies were downloaded in plain text format to establish a database.

### 2.2. Bibliometric and Visualization Analysis

**Figure 1:**
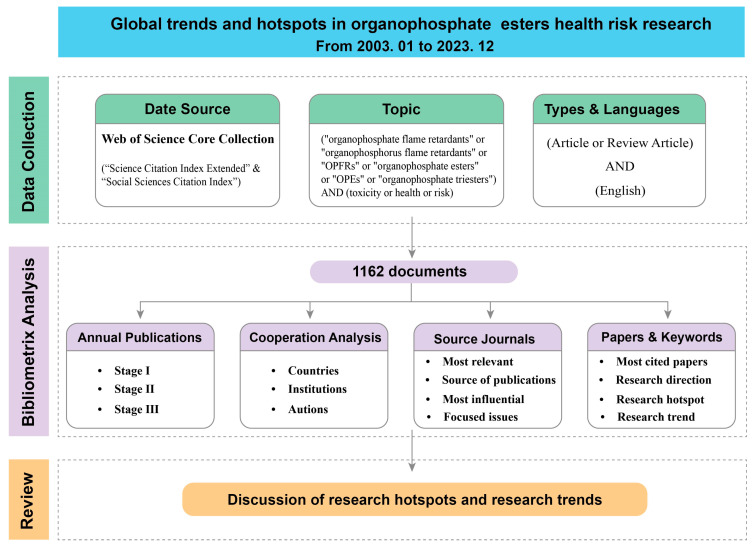
Schematic diagram of the research process.

The Bibliometrix package and CiteSpace software are commonly used analytical tools in bibliometrics. They were developed based on the R and Java programming languages, and each has its own strengths in bibliometric analysis and visualization [33,34]. The Bibliometrix package integrates numerous effective statistical algorithms with good flexibility and scalability for highly customized analysis and visualization. CiteSpace software has an intuitive user interface and a rich set of visualization tools that are widely used for burst detection analysis and time zone views. This study used the Bibliometrix package (version 4.1.4) and CiteSpace (version 6.1.R6) to conduct a longitudinal and integrative analysis of publication trends, countries, institutions, authors, source journals, important citations, and keywords to reveal the current state of the research and dynamics of the field. The flow of this work is shown in Figure 1.

## 3. Results and Discussion

### 3.1. Analysis of Temporal Trends in Publications

**Figure 2:**
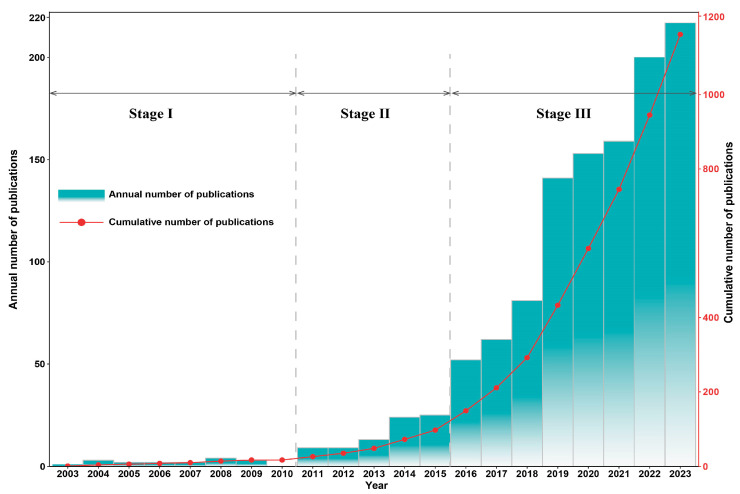
Chronological distribution of publications on health risk of OPFRs research from 2003 to 2023.

Statistical analysis of the number of published papers in a specific field is helpful for understanding the occurrence and development process of this research field [35]. There was a marked overall upward trend in the number of publications on the health risks of OPFRs during 2003–2023 (Figure 2). Specifically, this trend can be divided into three stages: (1) the preliminary exploration stage (2003–2010), (2) the steady development stage (2011–2015), and (3) the rapid development phase (2016–2023). (1) Preliminary exploration stage (2003–2010). During this period, the number of articles published per year fluctuated, and the average number of articles published per year was 2.1. Research on the health effects of OPFRs had just begun and was in its infancy. (2) Steady development stage (2011–2015). During this stage, global attention to OPFRs increased, and the number of articles published each year increased steadily, with an average of 16 articles per year. During this period, tetrabromodiphenyl ether, pentabromodiphenyl ether, and octabromodiphenyl ether were successively included in the Stockholm Convention on Persistent Organic Pollutants (POPs), and the global production and use of OPFRs as important alternatives to these compounds rapidly increased [6,7], which attracted the attention of many scholars who have begun to invest in this field. (3) Rapid development phase (2016–2023). Globally, the number of publications related to the health risks of OPFRs increased dramatically after 2016, from 52 in 2016 to 217 in 2023, with an average annual increase of 36.77%, and the average number of annual publications was 133.1. Per- and poly-fluoroalkyl substances (PFAS) are a new class of pollutants that have been the subject of considerable research fervor, with more than 4700 species used in the market and have attracted attention for more than 30 years [36]. The annual number of publications was obtained by searching for them in Web of Science in the same way. The cumulative number of publications of PFAS was higher than that of OPFRs during the same period (2016–2023), while the average annual growth rate of publications of OPFRs was higher than that of PFAS (26.28%). Details can be found in Table S1. This trend indicates that the health risks of OPFRs are becoming a popular research area that will receive more attention in the foreseeable future.

### 3.2. Analysis of Countries/Regions and Institutions

**Figure 3:**
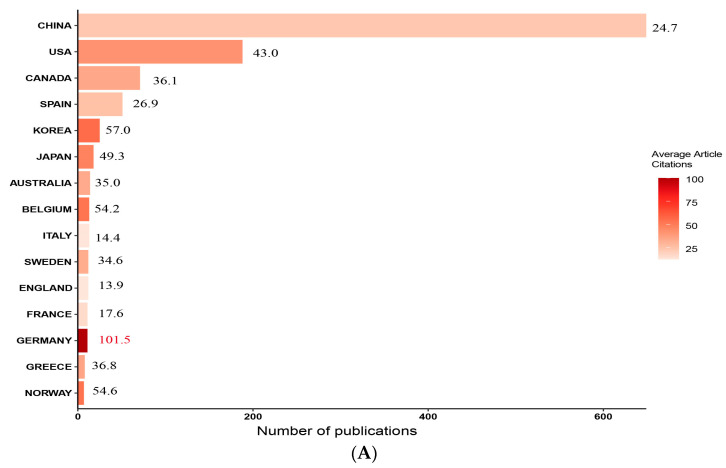

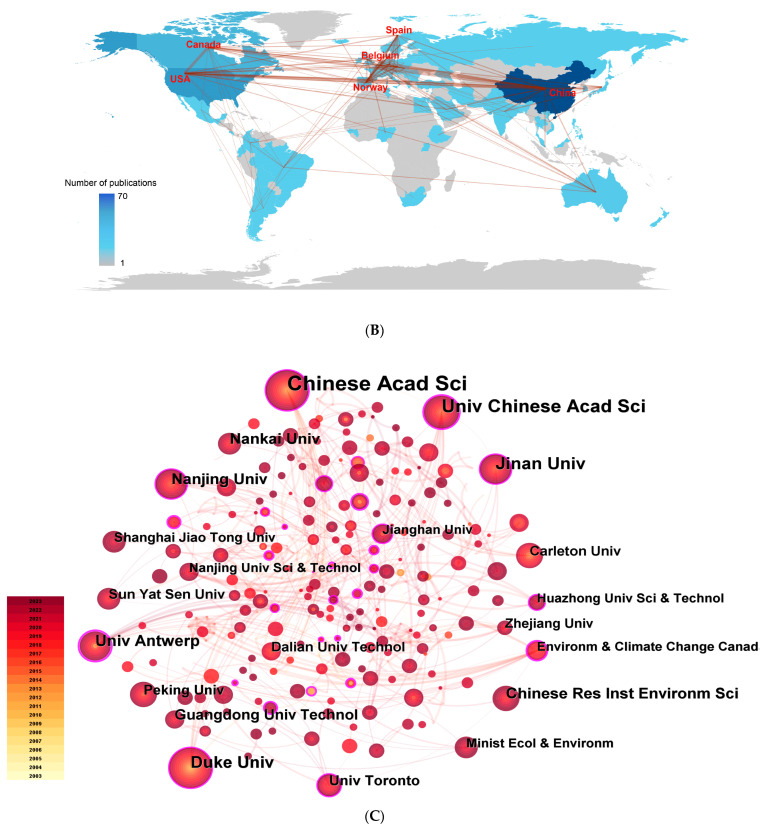
Country/region and institute network analysis. (**A**) The 15 countries with the greatest number of publications and average number of citations per year. The color and the text in front of the bar graph represent the average number of citations of the published literature in that country. (**B**) Country collaboration map based on the statistics of the countries to which all authors of an article belong. A darker color indicates more publications, and a denser connecting line indicates closer collaboration. (**C**) Research institution collaboration network. The nodes in the map represent institutions; larger nodes indicate a greater number of articles published by an institution and a closer relationship with other institutions. The connecting lines between the nodes indicate the co-occurrence relationship; line thickness indicates the intensity of co-occurrence, and a change in color from yellow to red indicates the time change from the early period to the recent period.

There were 70 countries/regions involved in this field during 2003–2023, with a total of 1162 publications. Attention to this field started late in China, with the earliest research published in 2009; however, the subsequent investment in research was relatively strong. By 2023, China accumulated 649 publications, ranking first in the world, followed by the United States and Canada, with 188 and 71 papers, respectively (Table S2 and Figure 3A). China and the United States, which have the highest number of publications, are also the world’s leading producers of OPFRs. A recent survey reported that there are about 367 OPFRs production plants in the world, of which 201 are located in China, 69 in the U.S., and the remaining 97 plants are scattered in 22 countries [37]. The high consumption of OPFRs seems to have contributed to its research fervor. The citations of a paper represent its academic impact [38]. The country with the highest average number of citations is Germany, which indicates that the average impact of its research achievements is high. China has the highest total number of citations with 16,012, but the average number of citations is 24.7, which indicates that although the number of publications in China far exceeds that of other countries, the quality of the literature varies, and the international influence of the research needs to be improved (Figure 3A). The country collaboration map (Figure 3B) visualizes the cooperation between countries worldwide in this field. The three countries with the largest number of articles, namely, China, the United States, and Canada, exhibit relatively close cooperation. Single-country publications (SCPs) and multiple-country publications (MCPs) are commonly used to measure the contribution of countries to cooperation at the international level [38,39]. While China, the United States, and Canada are important drivers in the field, there is a lack of collaborative research globally (MCP ratios of 0.21, 0.27, and 0.31, respectively). Details can be found in Table S2. Overall, international collaboration within the research field of the health risks of OPFRs is rare, and synergistic research in different geographic regions should be strengthened in the future to promote the development of the field.

The institutional collaboration network is shown in Figure 3C. The top 10 institutions in terms of publications are summarized in Table S3. Most of these institutions are from China, followed by the United States, Canada, and Belgium. The Chinese Academy of Sciences is the most prolific institution (168 papers), followed by the University of Chinese Academy of Sciences (82 papers), Jinan University (66 papers), Duke University (50 papers), and the University of Antwerp (43 papers). Centrality is a metric often used in CiteSpace to measure the importance of network mapping nodes [35] (Table S4). The centralities of the Chinese Academy of Sciences and the University of Antwerp are 0.40 and 0.23, respectively; these institutions are the core nodes in the network and, therefore, have greater influence in the field. The analysis of the institutional collaboration network shows that institutions with greater publication output tend to collaborate more extensively.

### 3.3. Author Analysis

**Figure 4:**
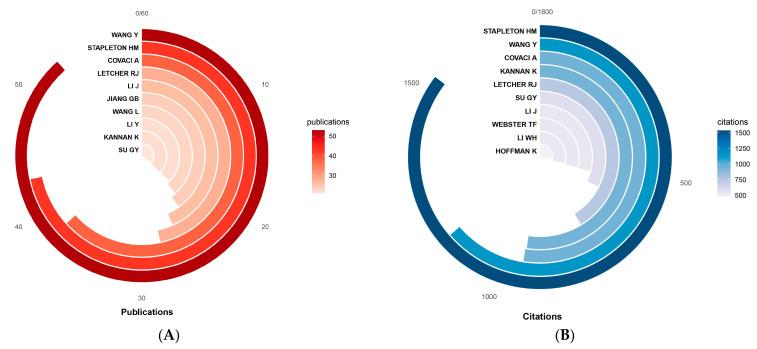

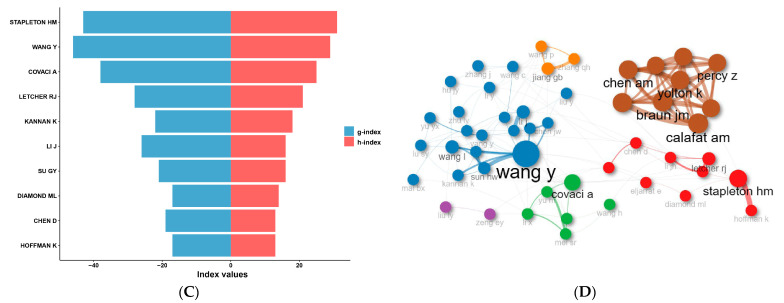
Author analysis. (**A**) Publications of authors (top 10); (**B**) citations of authors (top 10); (**C**) h-index and g-index of authors (top 10); (**D**) collaboration network of authors.

A total of 4111 authors were involved in this field between 2003 and 2023. The relationship between the amount of scientific literature and the number of authors in the field is in accordance with Lotka’s law [40], which means that the majority of authors (more than 90%) contribute a small number of studies (fewer than 4; Table S5 and Figure S2A). WANG Y published 53 publications and was the most prolific author, followed by STAPLETON HM (43 publications) and COVACI A (38 publications; Figure 4A). STAPLETON HM was the most highly cited author, with 1537 citations, followed by WANG Y (1147 citations), COVACI A (945 citations), and KANNAN K (941 citations; Figure 4B). The h-index and g-index are commonly used indicators of academic impact that synthesize the quantity and quality of research outputs, and each index has its own focus; they are used on a case-by-case basis [38,39] (Table S4). The h-index and g-index rankings of authors in the field are shown in Figure 4C. Combining the indicators, STAPLETON HM (h-index: 31; g-index: 43; publications: 43; citations: 3477; earliest publication: 2011) and WANG Y (h-index: 29; g-index: 46; publications: 53; citations: 2149; earliest publication: 2011) are the most influential authors. Both authors have focused on the field for more than 10 years, with WANG Y being relatively more active after 2017 (Figure S2B). In addition, COVACI A, LETCHER RJ, KANNAN K, and LI J are distinguished researchers in the field. Details can be found in Table S6.

The analysis of the authors’ cooperation network shows that a series of research teams with different focuses have been formed by high-level researchers in this field, and there is often cooperation among high-productivity authors (Figure 4D). Chinese scholars cooperate more closely in this field, while the cooperation among authors from other countries is relatively poor. In the future, scholars from different countries and different research directions should strengthen their cooperation and communication with each other to enhance the depth and breadth of research in this field.

### 3.4. Analysis of Source Journals

**Figure 5:**
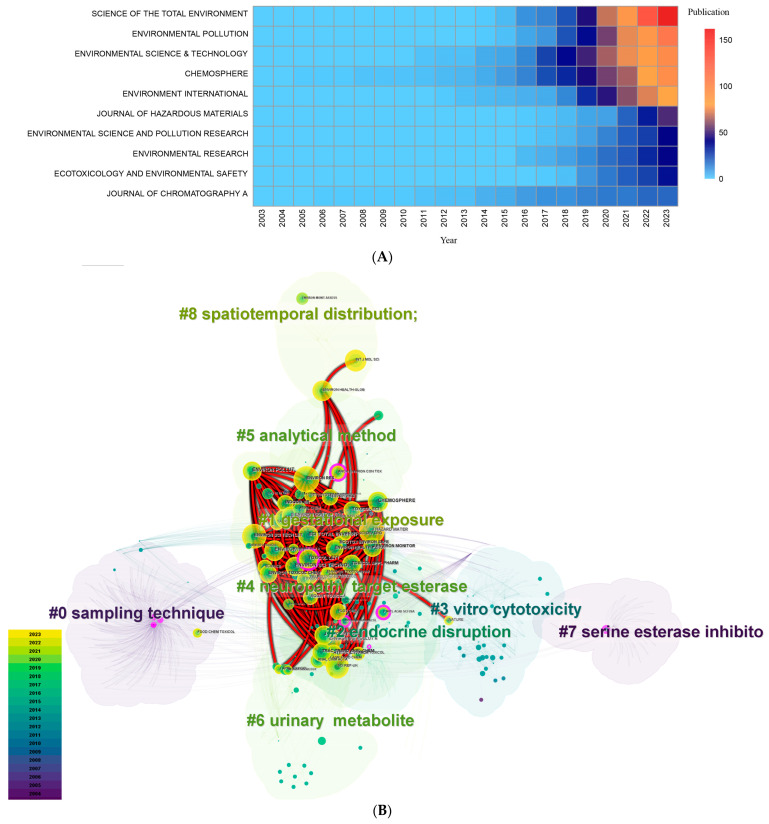
Source journal analysis. (**A**) Journal article volume over time. (**B**) Dual-map overlay of journal citation networks. The top panel shows the co-cited journal network from 2018–2023, the bottom panel shows the co-cited journal network from 2003–2023, and the red bold line represents the co-citation relationship between journals in the two periods.

A total of 173 academic journals published research papers related to the health risks of OPFRs during the period 2003–2023. Science of the Total Environment was the journal with the greatest number of publications, with a cumulative total of 162 papers, accounting for 13.94% of the total number of papers, followed by Environmental Pollution (112, 9.64%), Environmental Science and Technology (99, 8.52%) and Chemosphere (97, 8.35%), all with more than 90 publications. In terms of the literature source attribution of journals, Science of the Total Environment, Environmental Pollution, and the Journal of Hazardous Materials are more favorable to Chinese scholars, with a higher percentage of papers from China. Environmental Science and Technology, Environment International, and Environmental Research are relatively decentralized, featuring research from scholars in China, the U.S., Canada, and Spain (Figure S3A). The number of relevant studies in these journals sharply increased after 2016 (Figure 5A), which corresponds to the trend in the number of publications discussed in Section 3.1. Science of the Total Environment, Environmental Science and Technology, and the Journal of Hazardous Materials exhibited the fastest growth rates of related publications in the last three years, with an average annual growth of 31, 18.3, and 14.3 publications, respectively (Figure 5A); this result indicates that their attention to the field has increased dramatically. Combining the impact evaluation indexes of the h-index, g-index, citation frequency, and Bradford’s Law partition [34], Environmental Science and Technology, Science of the Total Environment, and Environment International were the most influential journals in the field during 2003–2023 (Table S7 and Figure S3B).

Journal co-citation maps for the last 20 years (2003–2023) and the last 5 years (2018–2023) were overlaid and analyzed to construct a time–journal biplot overlay network (Figure 5B). The cluster analysis of the journal literature showed that the main focuses of the journals on the health risks of OPFRs in the past 20 years could be summarized into eight main areas, namely, sampling technique, gestational exposure, endocrine disruption, in vitro cytotoxicity, neuropathy target esterase, analytical methods, urinary metabolites, serine esterase inhibition, and spatiotemporal distribution. The main topics covered in the literature published over the last 5 years were gestational exposure, endocrine disruption, in vitro cytotoxicity, neuropathy target esterase, and analytical methods. These findings suggest that developmental toxicity [41,42], endocrine disruption [16,43], and neurotoxicity [18,44] are the health risks of OPFRs that are of greatest concern; furthermore, determining the mechanism of toxicity [17,45] and evaluating exposure [10,46] are equally popular research directions.

The flow of discipline-specific knowledge at the journal level was obtained through dual-mapping overlay analysis of journals to intuitively reflect discipline-specific research trends [33]. In Figure S3C, the right side represents the distribution of cited journals, which serve as the information source; the left side represents the distribution of citing journals, which act as the convergence points. Disciplinary knowledge flows from right to left, forming an information flow. The information source provides theoretical and technical support for related research; the information flow represents the development progress and evolutionary direction of research at the macro level; and the convergence points indicate the hotspots and trends in the research field. The foundational knowledge in the field of OPFR health risks primarily originates from disciplines such as “Chemistry, Materials, Physics,” “Environmental, Toxicology, Nutrition,” “Molecular, Biology, Genetics,” and “Systems, Computing, Computer.” The information flow predominantly converges toward “Molecular Biology, Immunology,” “Veterinary, Animal, Science,” and “Ecology, Earth, Marine.” This result suggests that the next wave of research hotspots in this field may focus on molecular and biological immunology, animal science, and environmental ecology. This finding is consistent with the previous analysis, indicating that interest in the mechanisms of OPFRs is expected to continue to increase. The presence of multiple thick information flows in the figure indicates an increasing amount of interdisciplinary research, and the integration of knowledge across different disciplines is a significant direction for future studies.

### 3.5. Analysis of Highly Cited Literature

**Figure 6:**
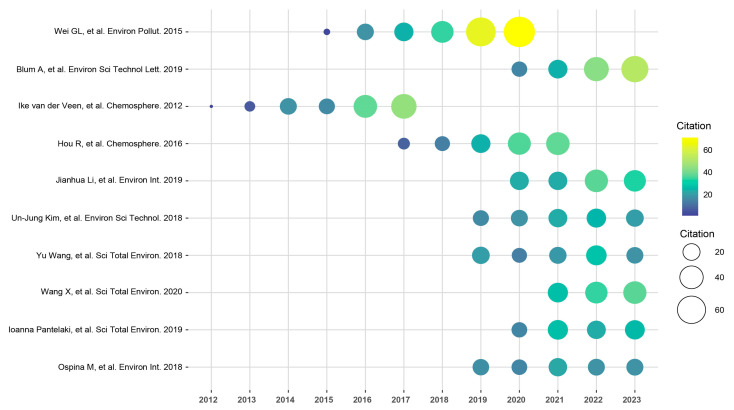
Temporal trend chart of literature citations. Literature reference citations: [7,9,47,48,51,52,53,54,55,56].

Table S8 summarizes the ten most-cited articles in the field of OPFR health risk research from 2003 to 2023, and Figure 6 displays their citation trends. Highly cited literature is a good metric for quickly understanding the current state of research in a field, and changes in the focus of these studies reflect the development trends of the research field. The study by Ike van der Veen et al. [47], frequently cited between 2012 and 2017, introduced the basic physicochemical properties of OPFRs; outlined the production and use of OPFRs before 2012; and highlighted the toxicity, analytical techniques, and contamination status of typical halogenated OPFRs such as tris(chloropropyl)phosphate (TCPP), tris(2-chloroethyl)phosphate (TCEP), and tris(1,3-dichloro-2-propyl)phosphate. Halogenated OPFRs were also the most frequently studied OPFRs before 2012. In contrast, the study by Wei GL et al. [7], frequently cited from 2015 to 2020, focused more on a broader exposure assessment of various OPFRs in environmental matrices, especially human exposure to OPFRs, and preliminarily evaluated the exposure levels of humans through air inhalation, dust inhalation, and dietary intake, suggesting that dust inhalation may be a more significant exposure pathway for humans. However, Li JH et al. [48] suggested that human exposure to OPFRs through diet is equally or more important. Their research focused on exposure to OPFRs in food, providing a detailed analysis of the contamination status of 30 types of OPFRs in various foods from countries worldwide. They observed that there are significant differences in OPFR levels between different countries or food categories; for example, grains and fats are the most contaminated products in China, while meat and fish are more severely contaminated in the United States. However, more than 22 OPFRs were frequently detected regardless of the sampling location or food group, and the problem of food contamination by OPFRs should not be ignored. The study by Wang X et al. [9], which has gained considerable attention in recent years, detailed the latest information on the detection methods, environmental occurrence, toxicity, and risk assessment of OPFRs in the relevant literature. They observed that although OPFRs in indoor dust are usually high, dietary intake is a more important exposure pathway because the consumption rate is much greater for humans (at least 1000 times greater). In fact, the bioavailability of OPFRs taken up by humans through different pathways has not yet been fully elucidated, and there are differences in the main exposure pathways for different OPFRs [49,50]; moreover, there is still substantial controversy about the mechanisms of OPFR exposure in humans, which requires further exploration. Research by Blum A et al. [51], which has gained the most attention in recent years, has continued to increase from 2020 to 2023. Blum A et al. questioned whether OPFRs were safer than PBDEs, and they conducted a detailed comparison of the two from multiple perspectives, such as environmental exposure status, toxicity testing, and the current state of epidemiological research. These authors believe that OPFRs are persistent mobile organic compounds (PMOCs), that their exposure levels in most environmental media continue to increase, that the potential exposure risk to populations has already far exceeded the peak levels during the use of PBDEs, that both halogenated and nonhalogenated OPFRs pose health risks, and that the extensive use of OPFRs requires wider attention and deeper research.

### 3.6. Keyword Analysis

#### 3.6.1. Analysis of Keyword Frequency and Clustering

**Figure 7:**
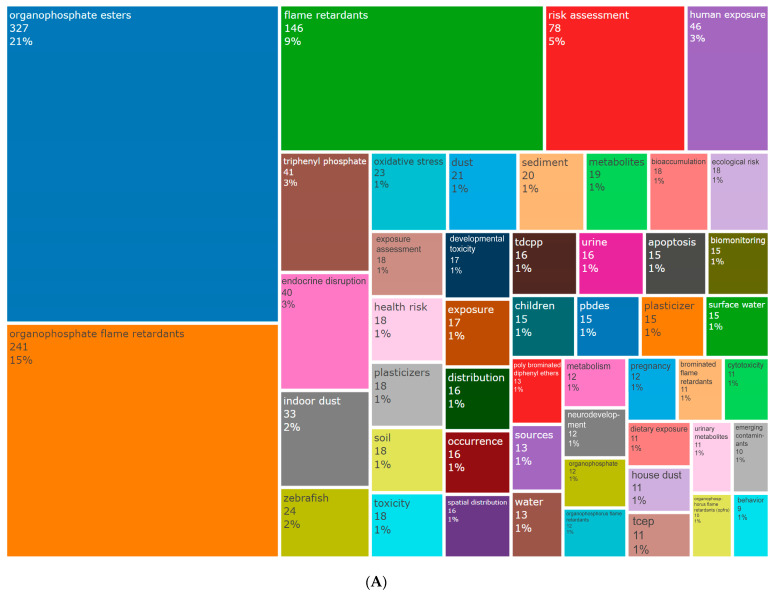

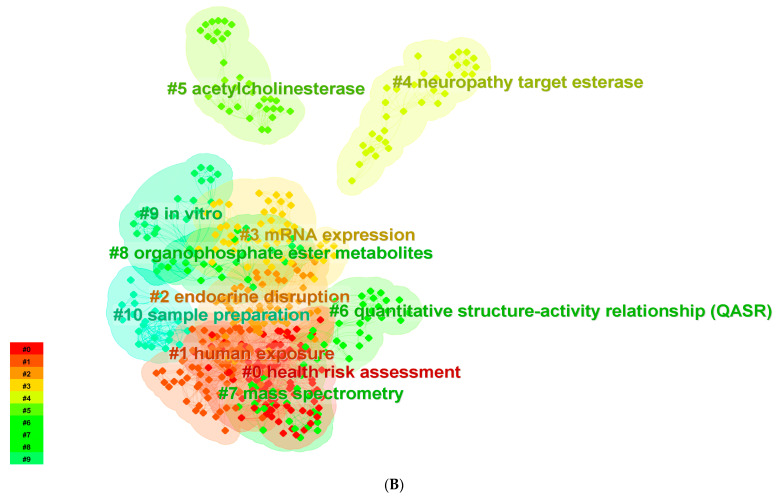
Keyword analysis. (**A**) Keyword dendrogram. The numbers in the color blocks indicate the frequency of keyword occurrence, and the percentage represents the importance of the keyword relative to the entire keyword set (measured based on term frequency-inverse document frequency). (**B**) Keyword clustering.

Keywords are a high-level summary of the content of studies, and analyzing the frequency and co-occurrence of keywords in the scientific literature can reveal the core themes of the research and help to identify important topics and trends in a particular field of study [57,58]. Publications related to the study of the health risks of OPFRs in the years 2003–2023 contained a total of 2692 author-supplied keywords. Of these, “organophosphate esters” (327 times), “organophosphate flame retardants” (241 times), “flame retardants” (146 times), “risk assessment” (78 times), and “human exposure” (46 times) were the five most frequent keywords (Figure 7A and Table S9). “Indoor dust” was the most frequently occurring keyword in the environmental media category; “zebrafish” was the most commonly used model organism; and “endocrine disruption” and “oxidative stress” were the most frequent keywords in terms of toxicity and mechanism.

The keywords were clustered using CiteSpace, and the labels were extracted using the log-likelihood ratio (LLR) algorithm; the results are shown in Figure 7B. Modularity and silhouette are important parameters for evaluating the clustering effect in the clustering map. The value interval of both indices is 0~1; the closer the value is to 1, the closer the connection between nodes in the cluster, and the better the clustering result (Table S4). A modality value >0.3 indicates that the clustering network structure is significant; a silhouette value >0.7 indicates that the results have high confidence [59]. In this study, the modality and silhouette values were 0.6355 and 0.8216, respectively, which indicates that the clusters were well structured and homogeneous and that the clustering results had a high degree of confidence. The cluster starts at 0, and the smaller the number is, the more members are included in the cluster; the major categories with more than 20 members were selected for analysis. Eleven major research directions were detected during the period of 2003–2023 (Figure 7B). The largest cluster was “health risk assessment”, followed by “human exposure”, “endocrine disruption”, “mRNA expression”, and “neuropathy target esterase”. Table S9 shows the details of each classification cluster and the main keywords used.

The clustering results indicated that from 2003 to 2023, research on the health risks of OPFRs primarily focused on three main aspects (Table S10): (1) Exposure assessment in various environmental media (Clusters 1, 7, and 10) mainly involved the use of gas chromatography, liquid chromatography, and mass spectrometry techniques for sample preparation and detection in air [60,61,62], water [63,64,65], soil [66,67], dust [68,69,70,71], human blood [72,73,74], and urine [75,76,77] to evaluate the spatiotemporal distribution of OPFRs and the exposure burden on organisms [78,79,80,81]. (2) Toxicity effects and mechanism research (Clusters 2, 3, 4, 5, and 9) on the health effects of OPFRs has primarily concentrated on neurotoxicity [17,18,44], developmental toxicity [19,20,41], and endocrine disruption effects [14,15,16] over the past two decades, exploring the associations, mechanisms, and pathways between OPFRs and various adverse health outcomes during in vivo and in vitro experiments using zebrafish [18,20], mice [82,83], chicken embryos [84], and various human cells [85,86]. (3) Research into the quantitative structure-activity relationships and metabolic mechanisms of OPFRs (Clusters 6 and 8) utilized quantitative structure-activity relationship (QSAR) models based on various molecular descriptors of OPFRs to assess and predict their fate and toxicity effects [17,45]. In approximately 2013, the metabolic processes and health effects of OPFRs and their metabolites gained increasing attention. OPFRs are primarily metabolized in vivo through O-dealkylation, hydroxylation, oxidative dechlorination, oxidation, and conjugation pathways, producing organophosphate diesters/di-alkyl phosphates (DAPs) and other products during phase I and phase II metabolic processes [87,88]. Recent studies have shown associations between these metabolites and various adverse health effects, such as preterm birth of fetuses [77], metabolic disorders in adults [15,76], semen quality [16], and uterine fibroids [89]; however, the underlying mechanisms remain unclear and warrant further investigation.

#### 3.6.2. Trend Analysis of Keywords

Since 2003, research related to health risk assessment (Cluster 0), human exposure (Cluster 1), and endocrine disruption (Cluster 2) of OPFRs has remained popular (Figure S4). Health risk assessment is a multidimensional and systematic term with strong and complex connectivity with other classifications. The likely reason for the continued research enthusiasm is the continuous deepening and improvement of exposure assessment techniques for OPFRs (Clusters 7 and 10) as well as studies of toxicity mechanisms at the molecular and cellular levels (Clusters 3, 4, and 9) during the past 20 years.

The keywords involved in the relevant studies from 2003 to 2023 were probed for burst strength, by which the changing trend of the research content in this field can be understood. A total of 36 keywords with high burst strength were identified, as shown in Table 1. The top 5 keywords in terms of burst strength include “indoor dust” (burst strength, 11.35), “mass spectrometry” (burst strength, 10.31), “dietary exposure” (burst strength, 8.51), “messenger RNA expression” (burst strength, 8.29), and “settled dust” (burst strength, 7.08). Keywords with burst strengths lasting through 2023 include “foodstuff”, “dietary exposure”, “nontarget screening”, “seasonal variation”, and “in vitro”.

Overall, during the preliminary exploration stage (2003–2010), there was limited understanding of the physicochemical properties and environmental fate patterns of OPFRs. Research at this time focused on the establishment and refinement of analytical techniques such as gas chromatography, liquid chromatography, and solid-phase extraction for environmental media such as water and air [90,91,92]. In the steady development stage (2011–2015), owing to advancements in analytical methods from the previous period, researchers began to widely identify and assess the environmental exposure of typical OPFRs such as triphenyl phosphate (TPhP), TDCPP, and TCEP and explored the endocrine disruption and neurotoxicity mechanisms of OPFRs through animal experiments using model organisms such as zebrafish and mice [7,93,94]. In the rapid development phase (2016–2023), significant improvements in mass spectrometry technology, including the development of high-resolution and nontargeted techniques, further enhanced exposure analysis. Researchers have conducted more comprehensive evaluations of the occurrence of OPFRs in various environmental media (sewage, air, particulate matter, food materials, human urine, etc.). During 2016–2023, 67 new OPFRs were discovered, most of which had (almost) 100% detection frequency in certain media [95]. Toxicity testing, population exposure assessment, metabolic pattern analysis, and safety evaluation of the numerous novel OPFRs are issues that urgently need to be addressed.

## 4. Key Findings and Future Directions

(1) Research on the health risks of OPFRs from 2003 to 2023 can be divided into three phases, with the field entering a rapid development stage after 2016, as the annual growth rate in the number of publications reached 34.4%. Global collaboration in this field is not extensive, with less than 30% of studies exhibiting international collaborative research despite China and the United States being the most prolific countries in terms of publications. The Chinese Academy of Sciences and the University of Antwerp are leading institutions in this area. After a comprehensive evaluation, STAPLETON HM and WANG Y were identified as the most influential researchers among the 4111 authors who contributed to the field.

(2) Papers related to research on the health risks of OPFRs from 2003–2023 were published in 173 academic journals. The majority of research in this field is published in high-impact journals, with Science of the Total Environment publishing the most papers. Combining multiple impact evaluation metrics, Environmental Science and Technology is the most influential journal in the field. The results of the journal cluster analysis suggest that the exposure evaluation and toxicity mechanism of OPFRs at multidisciplinary intersections will be an important focus for future journals.

(3) The results of the keyword analysis show that from 2003 to 2023, the research focus in this field gradually shifted from the distribution characteristics of environmental pollution by OPFRs to the exploration of the association with health effects and in-depth study of toxic effects; research is currently more focused on the evaluation of the exposure risk in the population and the exploration of the mechanism of health hazards. Future research will continue to focus on the comprehensive exposure evaluation of OPFRs in the population, exploring the mechanism of endocrine-disrupting effects and the health effects of metabolic processes and metabolites in vivo.

Although bibliometrics has macro, quantitative, and objective advantages in analyzing the research hotspots and development trends of a certain field, this study still has some limitations. First, only data from WOSCC were analyzed in this study. Although WOSCC contains mostly high-quality research papers, which are generally considered not to have a significant impact on the overall trend of the results, it is undeniable that the omission of databases such as Scopus and PubMed may lead to bias. Second, only research papers and review papers published in English were included in the study, which may introduce language bias and data omission. A more comprehensive bibliometric analysis in the field of the health risks of OPFRs should be based on mixed databases that are not limited by language, and we hope that in the future other researchers will be able to do this and compare the results.

## Figures and Tables

**Table 1:** Keyword burst table.

Keywords	Strength	Begin	End	2003–2023 *
gene	3.89	2004	2016	▂ ▃▃▃▃▃▃▃▃▃▃▃▃▃ ▂▂▂▂▂▂▂
organophosphate ester	3.80	2005	2015	▂▂ ▃▃▃▃▃▃▃▃▃▃▃ ▂▂▂▂▂▂▂▂
gas chromatography	5.44	2007	2018	▂▂▂▂ ▃▃▃▃▃▃▃▃▃▃▃▃ ▂▂▂▂▂
solid-phase extraction	6.09	2008	2017	▂▂▂▂▂ ▃▃▃▃▃▃▃▃▃▃ ▂▂▂▂▂▂
liquid chromatography	4.74	2008	2018	▂▂▂▂▂ ▃▃▃▃▃▃▃▃▃▃▃ ▂▂▂▂▂
indoor environment	4.37	2008	2018	▂▂▂▂▂ ▃▃▃▃▃▃▃▃▃▃▃ ▂▂▂▂▂
identification	5.45	2012	2018	▂▂▂▂▂▂▂▂▂ ▃▃▃▃▃▃▃ ▂▂▂▂▂
rat	3.24	2013	2016	▂▂▂▂▂▂▂▂▂▂ ▃▃▃▃ ▂▂▂▂▂▂▂
thyroid hormone	2.85	2013	2017	▂▂▂▂▂▂▂▂▂▂ ▃▃▃▃▃ ▂▂▂▂▂▂
neurotoxicity	2.56	2014	2018	▂▂▂▂▂▂▂▂▂▂▂ ▃▃▃▃▃ ▂▂▂▂▂
messenger RNA expression	8.29	2014	2017	▂▂▂▂▂▂▂▂▂▂▂ ▃▃▃▃ ▂▂▂▂▂▂
Extraction	4.48	2014	2017	▂▂▂▂▂▂▂▂▂▂▂ ▃▃▃▃ ▂▂▂▂▂▂
zebrafish embryos/larvae	2.24	2014	2016	▂▂▂▂▂▂▂▂▂▂▂ ▃▃▃ ▂▂▂▂▂▂▂
TDCPP	4.22	2014	2017	▂▂▂▂▂▂▂▂▂▂▂ ▃▃▃▃ ▂▂▂▂▂▂
particle	3.98	2014	2017	▂▂▂▂▂▂▂▂▂▂▂ ▃▃▃▃ ▂▂▂▂▂▂
developmental toxicity	2.13	2015	2017	▂▂▂▂▂▂▂▂▂▂▂▂ ▃▃▃ ▂▂▂▂▂▂
variability	3.74	2015	2017	▂▂▂▂▂▂▂▂▂▂▂▂ ▃▃▃ ▂▂▂▂▂▂
persistent organic pollutant	3.31	2015	2017	▂▂▂▂▂▂▂▂▂▂▂▂ ▃▃▃ ▂▂▂▂▂▂
mass spectrometry	10.31	2016	2018	▂▂▂▂▂▂▂▂▂▂▂ ▂▂ ▃▃▃ ▂▂▂▂▂
house dust	3.67	2016	2019	▂▂▂▂▂▂▂▂ ▂▂▂▂▂ ▃▃▃▃ ▂▂▂▂
environmental occurrence	5.48	2017	2019	▂▂▂▂▂▂▂▂▂▂ ▂▂▂▂ ▃▃▃ ▂▂▂▂
human exposure	4.82	2017	2019	▂▂▂▂▂▂▂▂▂▂▂ ▂▂▂ ▃▃▃ ▂▂▂▂
outdoor air	3.99	2017	2019	▂▂▂▂▂▂▂▂▂▂▂▂▂▂ ▃▃▃ ▂▂▂▂
halogenated flame retardant	3.56	2017	2020	▂▂▂▂▂▂▂▂▂▂▂▂▂▂ ▃▃▃▃ ▂▂▂
sewage treatment plant	3.36	2017	2018	▂▂▂▂▂▂▂▂▂▂▂▂▂▂ ▃▃ ▂▂▂▂▂
indoor dust	11.35	2018	2019	▂▂▂▂▂▂▂▂▂ ▂▂▂▂▂▂ ▃▃ ▂▂▂▂
settled dust	7.08	2018	2020	▂▂▂▂▂▂▂▂▂ ▂▂▂▂▂▂ ▃▃▃ ▂▂▂
in vitro metabolism	5.52	2018	2021	▂▂▂▂▂▂▂▂▂▂▂▂▂ ▂▂ ▃▃▃▃ ▂▂
diphenyl phosphate	5.35	2018	2021	▂ ▂▂▂▂▂▂▂▂▂▂▂▂▂▂ ▃▃▃▃ ▂▂
urinary metabolite	3.26	2019	2021	▂▂▂▂▂▂▂▂▂▂▂ ▂▂▂▂▂ ▃▃▃ ▂▂
sediment	6.16	2020	2023	▂▂▂▂▂▂▂▂▂▂▂▂ ▂▂▂▂▂ ▃▃▃▃
foodstuff	5.47	2020	2023	▂▂▂▂▂▂▂▂▂▂▂▂▂▂▂▂▂ ▃▃▃▃
dietary exposure	8.51	2021	2023	▂▂▂▂▂▂▂▂▂▂▂▂▂▂▂▂▂▂ ▃▃▃
nontarget screening	6.47	2021	2023	▂▂▂▂▂▂▂▂▂▂▂▂▂▂▂ ▂▂▂ ▃▃▃
seasonal variation	4.86	2021	2023	▂▂▂▂▂▂▂▂▂▂▂▂ ▂▂▂▂▂▂ ▃▃▃
in vitro	4.16	2021	2023	▂▂▂▂▂▂▂▂ ▂▂▂▂▂▂▂▂▂▂ ▃▃▃

*: The blue line indicates the timeline; dark blue indicates the beginning of the term; red indicates an outbreak of the term. The length of the color indicates the start year, end year, and duration of the outbreak.

## Data Availability

Not applicable.

## Supplementary Materials

The following supporting information can be downloaded at: https://www.mdpi.com/article/10.3390/toxics12060391/s1, Table S1: Annual Publication of Organophosphate flame retardant (OPFRs) and Per- and polyfluoroalkyl substances (PFAS) in Web of Science Core Collection (2016–2023); Table S2: The 15 most productive countries; Table S3: The10 most productive institutions; Table S4: Bibliometric parameters involved in the study [96,97,98]; Table S5: Author productivity through Lotka’s Law; Table S6: Top 50 authors overall (sort by h-index in descending order in the table); Table S7: Top 50 journals overall (sort by h-index in descending order in the table); Table S8: Top 10 most highly cited literature related to OPFRs health risk research; Table S9: Top 100 most frequent keywords; Table S10: Main clustering and internal keywords; Figure S1: Publication selection and flow chart of the research framework; Figure S2: Author analysis. (A) Lotka’s law fitting curve; (B) Top 10 author’s production over time; note: the size of the circle represents the number of articles published in the year, and the shade of the color represents the average number of citations per year; Figure S3: Source analysis. (A) Country-Journal-Author Sankey diagram; Note: The length of a rectangular node is proportional to the frequency of occurrence, and the width of the line between nodes is proportional to the number of connections; (B) Bradford’s law analysis based on the R-bibliometrix package. Gray represents the core area of the journal; (C) Discipline-journal dual-mapping overlay analysis. Different colors on the left side represent different types of citing journals, and different colors on the right side represent different types of cited journals; Figure S4: Keyword clustering—timeline diagram.

## References

[1] Patisaul H.B., Behl M., Birnbaum L.S., Blum A., Diamond M.L., Rojello Fernández S., Hogberg H.T., Kwiatkowski C.F., Page J.D., Soehl A. (2021). Beyond Cholinesterase Inhibition: Developmental Neurotoxicity of Organophosphate Ester Flame Retardants and Plasticizers. Environ. Health Perspect..

[2] Yang W., Zhao F., Fang Y., Li L., Li C., Ta N. (2018). 1H-Nuclear Magnetic Resonance Metabolomics Revealing the Intrinsic Relationships between Neurochemical Alterations and Neurobehavioral and Neuropathological Abnormalities in Rats Exposed to Tris(2-Chloroethyl)Phosphate. Chemosphere.

[3] Leong W.I., Lo O.L.I., Cheng F.T., Cheong W.M., Seak L.C.U. (2021). Using Recombinant Adhesive Proteins as Durable and Green Flame-Retardant Coatings. Synth. Syst. Biotechnol..

[4] Han F., Chen G., Tao G., Xu J., Zhang H., Zhang L., Li H., Zhao Y., Tian D., Kimura S.Y. (2022). Thyroid-Disrupting Effects Caused by Exposure to Alternative Flame Retardants from Groundwater Contamination in Rural Central China. Sci. Total Environ..

[5] Pantelaki I., Voutsa D. (2020). Occurrence, Analysis and Risk Assessment of Organophosphate Esters (OPEs) in Biota: A Review. Mar. Pollut. Bull..

[6] Van den Eede N., Ballesteros-Gómez A., Neels H., Covaci A. (2016). Does Biotransformation of Aryl Phosphate Flame Retardants in Blood Cast a New Perspective on Their Debated Biomarkers?. Environ. Sci. Technol..

[7] Wei G.-L., Li D.-Q., Zhuo M.-N., Liao Y.-S., Xie Z.-Y., Guo T.-L., Li J.-J., Zhang S.-Y., Liang Z.-Q. (2015). Organophosphorus Flame Retardants and Plasticizers: Sources, Occurrence, Toxicity and Human Exposure. Environ. Pollut..

[8] Global Flame Retardant Chemical Market Growth Analysis Report. https://www.bccresearch.com/market-research/chemicals/flame-retardant-chemicals-markets-report.html.

[9] Wang X., Zhu Q., Yan X., Wang Y., Liao C., Jiang G. (2020). A Review of Organophosphate Flame Retardants and Plasticizers in the Environment: Analysis, Occurrence and Risk Assessment. Sci. Total Environ..

[10] Zhang R., Li N., Li J., Zhao C., Luo Y., Wang Y., Jiang G. (2022). Percutaneous Absorption and Exposure Risk Assessment of Organophosphate Esters in Children’s Toys. J. Hazard. Mater..

[11] Yao C., Yang H., Li Y. (2021). A Review on Organophosphate Flame Retardants in the Environment: Occurrence, Accumulation, Metabolism and Toxicity. Sci. Total Environ..

[12] Admin O. The Proposition 65 List. https://oehha.ca.gov/proposition-65/proposition-65-list.

[13] Hoffman K., Sosa J.A., Stapleton H.M. (2017). Do Flame Retardant Chemicals Increase the Risk for Thyroid Dysregulation and Cancer?. Curr. Opin. Oncol..

[14] Zhang G., Meng L., Guo J., Guan X., Liu M., Han X., Li Y., Zhang Q., Jiang G. (2023). Exposure to Novel Brominated and Organophosphate Flame Retardants and Associations with Type 2 Diabetes in East China: A Case-Control Study. Sci. Total Environ..

[15] Ding E., Deng F., Fang J., Li T., Hou M., Liu J., Miao K., Yan W., Fang K., Shi W. (2023). Association between Organophosphate Ester Exposure and Insulin Resistance with Glycometabolic Disorders among Older Chinese Adults 60-69 Years of Age: Evidence from the China BAPE Study. Environ. Health Perspect..

[16] Siddique S., Farhat I., Kubwabo C., Chan P., Goodyer C.G., Robaire B., Chevrier J., Hales B.F. (2022). Exposure of Men Living in the Greater Montreal Area to Organophosphate Esters: Association with Hormonal Balance and Semen Quality. Environ. Int..

[17] Liu W., Luo D., Xia W., Tao Y., Wang L., Yu M., Hu L., Zhou A., Covaci A., Lin C. (2021). Prenatal Exposure to Halogenated, Aryl, and Alkyl Organophosphate Esters and Child Neurodevelopment at Two Years of Age. J. Hazard. Mater..

[18] Shi Q., Guo W., Shen Q., Han J., Lei L., Chen L., Yang L., Feng C., Zhou B. (2021). In Vitro Biolayer Interferometry Analysis of Acetylcholinesterase as a Potential Target of Aryl-Organophosphorus Flame-Retardants. J. Hazard. Mater..

[19] Luo D., Liu W., Wu W., Tao Y., Hu L., Wang L., Yu M., Zhou A., Covaci A., Xia W. (2021). Trimester-Specific Effects of Maternal Exposure to Organophosphate Flame Retardants on Offspring Size at Birth: A Prospective Cohort Study in China. J. Hazard. Mater..

[20] Tran C.M., Lee H., Lee B., Ra J.-S., Kim K.-T. (2021). Effects of the Chorion on the Developmental Toxicity of Organophosphate Esters in Zebrafish Embryos. J. Hazard. Mater..

[21] Bekele T.G., Zhao H., Yang J., Chegen R.G., Chen J., Mekonen S., Qadeer A. (2021). A Review of Environmental Occurrence, Analysis, Bioaccumulation, and Toxicity of Organophosphate Esters. Environ. Sci. Pollut. Res..

[22] Greaves A.K., Letcher R.J. (2017). A Review of Organophosphate Esters in the Environment from Biological Effects to Distribution and Fate. Bull. Environ. Contam. Toxicol..

[23] Bordons M., Ángeles Zulueta M. (1999). ^a^ Evaluación de La Actividad Científica a Través de Indicadores Bibliométricos. Rev. Esp. Cardiol..

[24] Muir D.C.G., Getzinger G.J., McBride M., Ferguson P.L. (2023). How Many Chemicals in Commerce Have Been Analyzed in Environmental Media? A 50 Year Bibliometric Analysis. Environ. Sci. Technol..

[25] Zhao W., Teng M., Zhang J., Wang K., Zhang J., Xu Y., Wang C. (2022). Insights into the Mechanisms of Organic Pollutant Toxicity to Earthworms: Advances and Perspectives. Environ. Pollut..

[26] Mekontchou O.Y., Zhenhua Z., Nkoh J.N., Ymele E., Usman M. (2024). A Systematic Review of Polycyclic Aromatic Hydrocarbon Pollution: A Combined Bibliometric and Mechanistic Analysis of Research Trend toward an Environmentally Friendly Solution. Sci. Total Environ..

[27] Chen Y., Lin M., Zhuang D. (2022). Wastewater Treatment and Emerging Contaminants: Bibliometric Analysis. Chemosphere.

[28] Kokol P., Blažun Vošner H., Završnik J. (2021). Application of Bibliometrics in Medicine: A Historical Bibliometrics Analysis. Health Inf. Libr. J..

[29] Durieux V., Gevenois P.A. (2010). Bibliometric Indicators: Quality Measurements of Scientific Publication. Radiology.

[30] Gu C., Wang Z., Pan Y., Zhu S., Gu Z. (2023). Tungsten-Based Nanomaterials in the Biomedical Field: A Bibliometric Analysis of Research Progress and Prospects. Adv. Mater..

[31] Jiang S., Liu Y., Zheng H., Zhang L., Zhao H., Sang X., Xu Y., Lu X. (2023). Evolutionary Patterns and Research Frontiers in Neoadjuvant Immunotherapy: A Bibliometric Analysis. Int. J. Surg. Lond. Engl..

[32] Zhang L., Zheng H., Jiang S.-T., Liu Y.-G., Zhang T., Zhang J.-W., Lu X., Zhao H.-T., Sang X.-T., Xu Y.-Y. (2024). Worldwide Research Trends on Tumor Burden and Immunotherapy: A Bibliometric Analysis. Int. J. Surg. Lond. Engl..

[33] Chen C. (2006). CiteSpace II: Detecting and Visualizing Emerging Trends and Transient Patterns in Scientific Literature. J. Am. Soc. Inf. Sci. Technol..

[34] Aria M., Cuccurullo C. (2017). *Bibliometrix*: An R-Tool for Comprehensive Science Mapping Analysis. J. Informetr..

[35] Zeng N., Sun J.-X., Liu C.-Q., Xu J.-Z., An Y., Xu M.-Y., Zhang S.-H., Zhong X.-Y., Ma S.-Y., He H.-D. (2024). Knowledge Mapping of Application of Image-Guided Surgery in Prostate Cancer: A Bibliometric Analysis (2013–2023). Int. J. Surg. Lond. Engl..

[36] Hu J., Lyu Y., Chen H., Cai L., Li J., Cao X., Sun W. (2023). Integration of Target, Suspect, and Nontarget Screening with Risk Modeling for per- and Polyfluoroalkyl Substances Prioritization in Surface Waters. Water Res..

[37] Huang J., Ye L., Fang M., Su G. (2022). Industrial Production of Organophosphate Flame Retardants (OPFRs): Big Knowledge Gaps Need to Be Filled?. Bull. Environ. Contam. Toxicol..

[38] He H., Liu C., Chen M., Guo X., Li X., Xiang Z., Liao F., Dong W. (2023). Effect of Dietary Patterns on Inflammatory Bowel Disease: A Machine Learning Bibliometric and Visualization Analysis. Nutrients.

[39] Xu D., Yin X., Zhou S., Jiang Y., Xi X., Sun H., Wang J. (2022). A Review on the Remediation of Microplastics Using Constructed Wetlands: Bibliometric, Co-Occurrence, Current Trends, and Future Directions. Chemosphere.

[40] Nicholls P.T. (1989). Bibliometric Modeling Processes and the Empirical Validity of Lotka’s Law. J. Am. Soc. Inf. Sci..

[41] Liu W., Luo D., Zhou A., Li H., Covaci A., Xu S., Mei S., Li Y. (2024). Prenatal Exposure to Organophosphate Esters and Growth Trajectory in Early Childhood. Sci. Total Environ..

[42] Yang W., Braun J.M., Vuong A.M., Percy Z., Xu Y., Xie C., Deka R., Calafat A.M., Ospina M., Burris H.H. (2023). Gestational Exposure to Organophosphate Esters and Infant Anthropometric Measures in the First 4 Weeks after Birth. Sci. Total Environ..

[43] Kojima H., Takeuchi S., Itoh T., Iida M., Kobayashi S., Yoshida T. (2013). In Vitro Endocrine Disruption Potential of Organophosphate Flame Retardants via Human Nuclear Receptors. Toxicology.

[44] Zhong X., Yu Y., Wang C., Zhu Q., Wu J., Ke W., Ji D., Niu C., Yang X., Wei Y. (2021). Hippocampal Proteomic Analysis Reveals the Disturbance of Synaptogenesis and Neurotransmission Induced by Developmental Exposure to Organophosphate Flame Retardant Triphenyl Phosphate. J. Hazard. Mater..

[45] Qiao Y., Feng C., Jin X., Yan Z., Feng W., Wang Y., Bai Y. (2024). Concentration Levels and Ecological Risk Assessment of Typical Organophosphate Esters in Representative Surface Waters of a Megacity. Environ. Res..

[46] Lee S., Jeong W., Kannan K., Moon H.-B. (2016). Occurrence and Exposure Assessment of Organophosphate Flame Retardants (OPFRs) through the Consumption of Drinking Water in Korea. Water Res..

[47] van der Veen I., de Boer J. (2012). Phosphorus Flame Retardants: Properties, Production, Environmental Occurrence, Toxicity and Analysis. Chemosphere.

[48] Li J., Zhao L., Letcher R.J., Zhang Y., Jian K., Zhang J., Su G. (2019). A Review on Organophosphate Ester (OPE) Flame Retardants and Plasticizers in Foodstuffs: Levels, Distribution, Human Dietary Exposure, and Future Directions. Environ. Int..

[49] Tokumura M., Seo M., Wang Q., Miyake Y., Amagai T., Makino M. (2019). Dermal Exposure to Plasticizers in Nail Polishes: An Alternative Major Exposure Pathway of Phosphorus-Based Compounds. Chemosphere.

[50] Xu F., Giovanoulis G., van Waes S., Padilla-Sanchez J.A., Papadopoulou E., Magnér J., Haug L.S., Neels H., Covaci A. (2016). Comprehensive Study of Human External Exposure to Organophosphate Flame Retardants via Air, Dust, and Hand Wipes: The Importance of Sampling and Assessment Strategy. Environ. Sci. Technol..

[51] Blum A., Behl M., Birnbaum L.S., Diamond M.L., Phillips A., Singla V., Sipes N.S., Stapleton H.M., Venier M. (2019). Organophosphate Ester Flame Retardants: Are They a Regrettable Substitution for Polybrominated Diphenyl Ethers?. Environ. Sci. Technol. Lett..

[52] Hou R., Xu Y., Wang Z. (2016). Review of OPFRs in Animals and Humans: Absorption, Bioaccumulation, Metabolism, and Internal Exposure Research. Chemosphere.

[53] Kim U.-J., Kannan K. (2018). Occurrence and Distribution of Organophosphate Flame Retardants/Plasticizers in Surface Waters, Tap Water, and Rainwater: Implications for Human Exposure. Environ. Sci. Technol..

[54] Wang Y., Sun H., Zhu H., Yao Y., Chen H., Ren C., Wu F., Kannan K. (2018). Occurrence and Distribution of Organophosphate Flame Retardants (OPFRs) in Soil and Outdoor Settled Dust from a Multi-Waste Recycling Area in China. Sci. Total Environ..

[55] Pantelaki I., Voutsa D. (2019). Organophosphate Flame Retardants (OPFRs): A Review on Analytical Methods and Occurrence in Wastewater and Aquatic Environment. Sci. Total Environ..

[56] Ospina M., Jayatilaka N.K., Wong L.-Y., Restrepo P., Calafat A.M. (2018). Exposure to Organophosphate Flame Retardant Chemicals in the U.S. General Population: Data from the 2013-2014 National Health and Nutrition Examination Survey. Environ. Int..

[57] Chen C. (2020). A Glimpse of the First Eight Months of the COVID-19 Literature on Microsoft Academic Graph: Themes, Citation Contexts, and Uncertainties. Front. Res. Metr. Anal..

[58] Wang Y., Bai J., Zhang L., Liu H., Wang W., Liu Z., Zhang G. (2023). Advances in Studies on the Plant Rhizosphere Microorganisms in Wetlands: A Visualization Analysis Based on CiteSpace. Chemosphere.

[59] Chen C., Song M. (2019). Visualizing a Field of Research: A Methodology of Systematic Scientometric Reviews. PLoS ONE.

[60] Kurt-Karakus P., Alegria H., Birgul A., Gungormus E., Jantunen L. (2018). Organophosphate Ester (OPEs) Flame Retardants and Plasticizers in Air and Soil from a Highly Industrialized City in Turkey. Sci. Total Environ..

[61] Li J., Xie Z., Mi W., Lai S., Tian C., Emeis K.-C., Ebinghaus R. (2017). Organophosphate Esters in Air, Snow, and Seawater in the North Atlantic and the Arctic. Environ. Sci. Technol..

[62] Liu K., Xiao H., Zhang Y., He H., Li S., Yang S., Li H. (2023). Gas-Particle Partitioning of Organophosphate Esters in Indoor and Outdoor Air and Its Implications for Individual Exposure. Environ. Int..

[63] Wang T., He Z.-X., Yang J., Wu L., Qiu X.-W., Bao L.-J., Zeng E.Y. (2022). Riverine Transport Dynamics of PBDEs and OPFRs within a Typical E-Waste Recycling Zone: Implications for Sink-Source Interconversion. Water Res..

[64] Wang X., Zhu L., Zhong W., Yang L. (2018). Partition and Source Identification of Organophosphate Esters in the Water and Sediment of Taihu Lake, China. J. Hazard. Mater..

[65] Lin L., Huang Y., Wang P., Chen C.C., Qian W., Zhu X., Xu X. (2023). Environmental Occurrence and Ecotoxicity of Aquaculture-Derived Plastic Leachates. J. Hazard. Mater..

[66] You J., Chen Z.-M., Hou X.-Y., Guo J.-S., Wang C.-C., Gao J.-M. (2022). Occurrence, Potential Sources and Risks of Organophosphate Esters in the High-Elevation Region, Tibet, China. Sci. Total Environ..

[67] Yan Z., Feng C., Leung K.M.Y., Luo Y., Wang J., Jin X., Wu F. (2023). Insights into the Geographical Distribution, Bioaccumulation Characteristics, and Ecological Risks of Organophosphate Esters. J. Hazard. Mater..

[68] Zhao L., Zhang Y., Deng Y., Jian K., Li J., Ya M., Su G. (2020). Traditional and Emerging Organophosphate Esters (OPEs) in Indoor Dust of Nanjing, Eastern China: Occurrence, Human Exposure, and Risk Assessment. Sci. Total Environ..

[69] Tao F., Sellström U., de Wit C.A. (2019). Organohalogenated Flame Retardants and Organophosphate Esters in Office Air and Dust from Sweden. Environ. Sci. Technol..

[70] Vykoukalová M., Venier M., Vojta Š., Melymuk L., Bečanová J., Romanak K., Prokeš R., Okeme J.O., Saini A., Diamond M.L. (2017). Organophosphate Esters Flame Retardants in the Indoor Environment. Environ. Int..

[71] Chen Y., Cao Z., Covaci A., Li C., Cui X. (2019). Novel and Legacy Flame Retardants in Paired Human Fingernails and Indoor Dust Samples. Environ. Int..

[72] Hou M., Fang J., Shi Y., Tang S., Dong H., Liu Y., Deng F., Giesy J.P., Godri Pollitt K.J., Cai Y. (2021). Exposure to Organophosphate Esters in Elderly People: Relationships of OPE Body Burdens with Indoor Air and Dust Concentrations and Food Consumption. Environ. Int..

[73] Hou M., Shi Y., Jin Q., Cai Y. (2020). Organophosphate Esters and Their Metabolites in Paired Human Whole Blood, Serum, and Urine as Biomarkers of Exposure. Environ. Int..

[74] Liu Y., Li Y., Dong S., Han L., Guo R., Fu Y., Zhang S., Chen J. (2021). The Risk and Impact of Organophosphate Esters on the Development of Female-Specific Cancers: Comparative Analysis of Patients with Benign and Malignant Tumors. J. Hazard. Mater..

[75] He C., Toms L.-M.L., Thai P., Van den Eede N., Wang X., Li Y., Baduel C., Harden F.A., Heffernan A.L., Hobson P. (2018). Urinary Metabolites of Organophosphate Esters: Concentrations and Age Trends in Australian Children. Environ. Int..

[76] Luo K., Zhang R., Aimuzi R., Wang Y., Nian M., Zhang J. (2020). Exposure to Organophosphate Esters and Metabolic Syndrome in Adults. Environ. Int..

[77] Hoffman K., Stapleton H.M., Lorenzo A., Butt C.M., Adair L., Herring A.H., Daniels J.L. (2018). Prenatal Exposure to Organophosphates and Associations with Birthweight and Gestational Length. Environ. Int..

[78] Chen S.-C., Tao F., Liu W., Wang X., Ding J., Zhang Z., Ma D. (2023). Emerging and Traditional Organophosphate Esters in Office Air from Hangzhou, East China: Seasonal Variations, Influencing Factors and Human Exposure Assessment. Environ. Int..

[79] Li J., Tang J., Mi W., Tian C., Emeis K.-C., Ebinghaus R., Xie Z. (2018). Spatial Distribution and Seasonal Variation of Organophosphate Esters in Air above the Bohai and Yellow Seas, China. Environ. Sci. Technol..

[80] Li W., Yuan Y., Wang S., Liu X. (2023). Occurrence, Spatiotemporal Variation, and Ecological Risks of Organophosphate Esters in the Water and Sediment of the Middle and Lower Streams of the Yellow River and Its Important Tributaries. J. Hazard. Mater..

[81] Zhang Y., Wu M., Xu M., Hu P., Xu X., Liu X., Cai W., Xia J., Wu D., Xu X. (2022). Distribution of Flame Retardants among Indoor Dust, Airborne Particles and Vapour Phase from Beijing: Spatial-Temporal Variation and Human Exposure Characteristics. Environ. Int..

[82] Macari S., Rock K.D., Santos M.S., Lima V.T.M., Szawka R.E., Moss J., Horman B., Patisaul H.B. (2020). Developmental Exposure to the Flame Retardant Mixture Firemaster 550 Compromises Adult Bone Integrity in Male but Not Female Rats. Int. J. Mol. Sci..

[83] Zhang Q., Yu C., Fu L., Gu S., Wang C. (2020). New Insights in the Endocrine Disrupting Effects of Three Primary Metabolites of Organophosphate Flame Retardants. Environ. Sci. Technol..

[84] Kanda K., Ito S., Koh D.-H., Kim E.-Y., Iwata H. (2021). Effects of Tris(2-Chloroethyl) Phosphate Exposure on Chicken Embryos in a Shell-Less Incubation System. Ecotoxicol. Environ. Saf..

[85] Ji X., Li N., Ma M., Rao K., Wang Z. (2020). In Vitro Estrogen-Disrupting Effects of Organophosphate Flame Retardants. Sci. Total Environ..

[86] Li Z., Tang X., Zhu L., Qi X., Cao G., Lu G. (2020). Cytotoxic Screening and Transcriptomics Reveal Insights into the Molecular Mechanisms of Trihexyl Phosphate-Triggered Hepatotoxicity. Environ. Sci. Technol..

[87] Li J., Liu Y., Meng W., Su G. (2024). Biotransformation of Organophosphate Diesters Characterized via In Vitro Metabolism and In Vivo Screening. Environ. Sci. Technol..

[88] Wang X., Zhao X., Shi D., Dong Z., Zhang X., Liang W., Liu L., Wang X., Wu F. (2023). Integrating Physiologically Based Pharmacokinetic Modeling-Based Forward Dosimetry and in Vitro Bioassays to Improve the Risk Assessment of Organophosphate Esters on Human Health. Environ. Sci. Technol..

[89] Lee G., Kim S., Bastiaensen M., Malarvannan G., Poma G., Caballero Casero N., Gys C., Covaci A., Lee S., Lim J.-E. (2020). Exposure to Organophosphate Esters, Phthalates, and Alternative Plasticizers in Association with Uterine Fibroids. Environ. Res..

[90] Martínez-Carballo E., González-Barreiro C., Sitka A., Scharf S., Gans O. (2007). Determination of Selected Organophosphate Esters in the Aquatic Environment of Austria. Sci. Total Environ..

[91] Marklund A., Andersson B., Haglund P. (2005). Organophosphorus Flame Retardants and Plasticizers in Swedish Sewage Treatment Plants. Environ. Sci. Technol..

[92] Stapleton H.M., Klosterhaus S., Eagle S., Fuh J., Meeker J.D., Blum A., Webster T.F. (2009). Detection of Organophosphate Flame Retardants in Furniture Foam and U.S. House Dust. Environ. Sci. Technol..

[93] Androutsopoulos V.P., Kanavouras K., Tsatsakis A.M. (2011). Role of Paraoxonase 1 (PON1) in Organophosphate Metabolism: Implications in Neurodegenerative Diseases. Toxicol. Appl. Pharmacol..

[94] Tian Y.X., Chen H.Y., Ma J., Liu Q.Y., Qu Y.J., Zhao W.H. (2023). A Critical Review on Sources and Environmental Behavior of Organophosphorus Flame Retardants in the Soil: Current Knowledge and Future Perspectives. J. Hazard. Mater..

[95] Ye L., Li J., Gong S., Herczegh S.M., Zhang Q., Letcher R.J., Su G. (2023). Established and Emerging Organophosphate Esters (OPEs) and the Expansion of an Environmental Contamination Issue: A Review and Future Directions. J. Hazard. Mater..

[96] Hirsch J.E. (2005). An Index to Quantify an Individual’s Scientific Research Output. Proc. Natl. Acad. Sci. USA.

[97] Egghe L. (2006). Theory and Practise of the g-Index. SCIENTOMETRICS.

[98] Newman M.E.J. (2006). Modularity and Community Structure in Networks. Proc. Natl. Acad. Sci. USA.

