# Supplementary material for: Global Trends and Hotspots in Research on the Health Risks of Organophosphate Flame Retardants: A Bibliometric and Visual Analysis
Source: Toxics. 2024 May 27;12(6):391. doi: 10.3390/toxics12060391 (PMC11209454; doi:10.3390/toxics12060391)
Supplement: Supplementary file 1 [file toxics-12-00391-s001.zip › toxics-3001017-supplementary.pdf]

Supporting Information for

Global Trends and Hotspots in Research on the Health Risks of Organophosphate Flame Retardants: A Bibliometric and Visual Analysis

Zhiyuan Du [1], Yuanyuan Ruan [2,3], Jiabin Chen [1], Jian Fang [1], Shuo Xiao [4], Yewen Shi [5,*] and Weiwei Zheng [1,6,*]

[1] Key Laboratory of the Public Health Safety, Ministry of Education, Department of Environmental Health, School of Public Health, Fudan University, Shanghai 200032, China; duzhiyuan0422@163.com (Z.D.); 22301020036@m.fudan.edu.cn (J.C.); 22301020015@m.fudan.edu.cn (J.F.)

[2] NHC Key Laboratory of Glycoconjugates Research, School of Basic Medical Sciences, Fudan University, Shanghai 200032, China; yuanyuanruan@fudan.edu.cn

[3] Department of Biochemistry and Molecular Biology, School of Basic Medical Sciences, Fudan University, Shanghai 200032, China

[4] Department of Pharmacology and Toxicology, Ernest Mario School of Pharmacy, Environmental and
Occupational Health Sciences Institutes, Rutgers University, Piscataway, NJ 08854, USA; sx106@pharmacy.rutgers.edu

[5] Shanghai Municipal Center for Disease Control and Prevention, Shanghai 200336, China

[6] Center for Water and Health, School of Public Health, Fudan University, Shanghai 200032, China

* Correspondence: shiyewen@scdc.sh.cn (Y.S.); weiweizheng@fudan.edu.cn (W.Z.)

Table S1. Annual Publication of Organophosphate flame retardant (OPFRs) and Per- and polyfluoroalkyl substances (PFAS) in Web of Science Core Collection (2016-2023)

Year	Organophosphate flame retardant (OPFRs)		Per- and polyfluoroalkyl substances (PFAS)	
	Cumulative publications	Annual growth rate	Cumulative publications	Annual growth rate
2016	149	53.61%	690	22.34%
2017	211	41.61%	828	20.00%
2018	292	38.39%	1019	23.07%
2019	433	48.29%	1286	26.20%
2020	586	35.33%	1687	31.18%
2021	745	27.13%	2217	31.42%
2022	945	26.85%	2874	29.63%
2023	1162	23.00%	3633	26.41%

Table S2. The 15 most productive countries

Country	Publications	SCP[a]	MCP[b]	MCP_Ratio	Total citation	Average Article Citations	Year[c]
CHINA	649	513	136	0.21	16012	24.7	2009
USA	188	137	51	0.27	8082	43.0	2003
CANADA	71	49	22	0.31	2560	36.1	2011
SPAIN	51	32	19	0.37	1374	26.9	2008
KOREA	25	19	6	0.24	1425	57.0	2004
JAPAN	18	10	8	0.44	888	49.3	2004
AUSTRALIA	14	6	8	0.57	490	35.0	2014
BELGIUM	13	4	9	0.69	705	54.2	2011
ITALY	13	11	2	0.15	187	14.4	2005
SWEDEN	12	9	3	0.25	415	34.6	2012
ENGLAND	12	5	7	0.58	167	13.9	2005
FRANCE	11	8	3	0.27	194	17.6	2008
GERMANY	11	6	5	0.46	1117	101.5	2008
GREECE	8	8	0	/	294	36.8	2019
NORWAY	7	2	5	0.71	382	54.6	2014

[a] Single Country Publications, indicating that the corresponding authors of the article are from the same country;

[b] Multiple Country Publications, indicating that the corresponding author of the article is from multiple countries, that is, there is cooperation between countries;

[c] The year in which the relevant paper was first published in that country;

Table S3. The10 most productive institutions

Rank	Institution	Publications	Centrality	Year[a]
1	Chinese Academy of Sciences	168	0.40	2009
2	University of Chinese Academy of Sciences	82	0.05	2015
3	Jinan University	66	0.12	2016
4	Duke University	50	0.16	2011
5	Antwerp University	43	0.23	2011
6	Chinese Research Academy of Environmental Science	40	0.04	2018
7	Nanjing University	37	0.07	2014
8	Nankai University	37	0.02	2018
9	Guangdong University of Technology	30	0.00	2019
10	Toronto University	28	0.14	2018

[a] The year in which the relevant paper was first published in that institution;

Table S4. Bibliometric parameters involved in the study.

Items	Equations	Parameter illustrations	Purposes	References
Centrality (C_i)	$C_i = \sum_{i \neq j \neq k} \frac{\rho_{jk}(i)}{\rho_{jk}}$	ρ_{jk}: the number of shortest paths between node *j* and node *k*;<br>$\rho_{jk}(i)$: the number of paths passing through node *i*.	To quantify the importance of nodes in the bibliometric network.	1
h-index	There is no single "formula" for direct calculation, but rather a statistical and sorting process to derive the indicator.	The h-index can be defined as if a person/journal has N papers that have been cited at least N times each, then N is equal to the h-index.	To evaluate academic impact by combining the quantity and quality of academic outputs.	2
g-index	There is no single "formula" for direct calculation, but rather a statistical and sorting process to derive the indicator.	Given a set of articles in descending order of their number of citations, the G-index is the largest paper order g that has been cumulatively cited at least g^2 times, while the cumulative number of citations corresponding to the (g+1)th order paper will be less than $(g+1)^2$.	To evaluate academic impact by combining the quantity and quality of academic outputs.	3
Modularity (Q) : clustering module value	$Q = \sum_{i=1}^{n} (\frac{I_i}{E_i} - \left(\frac{2I_i + O_i}{2E_i}\right)^2)$	I_i: the number of links between two nodes both in the same cluster *i*;<br>*Oi*: the number of links between one node in the cluster *i* and another node not in the cluster *i*;<br>E_i: the total count of links within the cluster *I*;<br>*n*: the amount of clusters.	To judge whether the construction of clusters is remarkable. Generally, when the *Q* value is above 0.3, the clustering has a remarkable structure.	4
Silhouette coefficient (S) : average contour value of clustering	$S = \frac{\sum_{i=1}^{n} \frac{b_i - a_i}{\max(a_i, b_i)}}{n}$	a_i: the average Euclidean distance of node i to other nodes in the same cluster;<br>*bi*: the minimum average distance between node *i* and other nodes in different clusters.<br>*n*: same as Equ. 2.	To judge whether the clustering is reliable and credible. Generally, if the *S* value is above 0.5, the clustering is reasonable; If the S value is above 0.7, the clustering is convincing.	5

Table S5. Author productivity through Lotka's Law

Documents written	Number of Authors	Proportion of Authors
1	2731	0.664
2	662	0.161
3	284	0.069
4	141	0.034
5	83	0.02
6	49	0.012
7	39	0.009
8	23	0.006
9	21	0.005
10	17	0.004
11	10	0.002
12	14	0.003
13	8	0.002
14	4	0.001
15	2	0.000
16	2	0.000
17	3	0.001
18	4	0.001
19	2	0.000
20	2	0.000
21	1	0.000
22	2	0.000
23	1	0.000
24	1	0.000
26	1	0.000
28	1	0.000
38	1	0.000
43	1	0.000
53	1	0.000

Table S6. Top 50 authors overall (sort by h-index in descending order in the table).

Authors	h_index	g_index	Citations	Publications	Publication Year_start
STAPLETON HM	31	43	1537	43	2011
WANG Y	29	46	945	53	2011
COVACI A	25	38	1145	38	2011
LETCHER RJ	21	28	941	28	2011
KANNAN K	18	22	742	22	2016
SU GY	16	21	528	21	2014
LI J	16	26	421	26	2014
DIAMOND ML	14	17	584	17	2018
HOFFMAN K	13	17	530	17	2014
LI Y	13	22	481	22	2017
MAI BX	13	18	264	18	2015
CHEN D	13	19	387	19	2011
ZHAO HX	13	14	346	14	2017
JIANG GB	12	24	523	24	2016
ZHANG Y	12	20	373	20	2016
CHEN JW	12	13	354	13	2017
LIU XT	12	15	270	15	2018
ELJARRAT E	12	18	241	18	2016
WANG L	12	20	308	23	2017
YANG Y	12	20	290	20	2014
WEBSTER TF	11	11	329	11	2011
ZHANG G	11	17	275	17	2016
ZHANG Q	11	18	375	18	2017
JANTUNEN LM	11	11	345	11	2018
VAN DEN EEDE N	10	10	323	10	2012
VENIER M	10	11	320	11	2015
LI JH	10	14	249	14	2017
SUN HW	10	16	266	16	2018
CALAFAT AM	10	19	265	19	2017
ZENG EY	10	12	190	12	2016
OSPINA M	10	18	361	18	2018
LI WH	9	10	361	10	2016
XIE ZY	9	9	363	9	2015
YAO YM	9	14	233	14	2018

HU JY	9	12	233	12	2016
ZHU HK	9	10	229	10	2018
LIU LY	9	12	229	12	2015
WANG P	9	12	229	12	2015
YIN H	9	11	198	11	2019
CRUMP D	8	9	256	9	2012
MI WY	8	9	261	9	2015
MOON HB	8	9	333	9	2013
MEEKER JD	8	11	234	11	2013
SHI YL	8	10	240	10	2016
CAI YQ	8	9	182	9	2016
MUELLER JF	8	10	224	10	2015
SALAMOVA A	8	8	326	8	2015
WANG XY	8	10	285	10	2017
YU ZQ	8	9	317	9	2014
HE C	8	9	275	9	2017

Table S7. Top 50 journals overall (sort by h-index in descending order in the table).

Journal	h_index	g_index	Citations	Publications	Bradford Law_Zone	Publication Year_start
ENVIRONMENTAL SCIENCE & TECHNOLOGY	42	71	5285	99	Zone 1	2011
ENVIRONMENT INTERNATIONAL	41	62	3957	82	Zone 2	2012
SCIENCE OF THE TOTAL ENVIRONMENT	37	60	4627	162	Zone 1	2014
ENVIRONMENTAL POLLUTION	34	53	3338	112	Zone 1	2014
CHEMOSPHERE	32	50	2880	97	Zone 1	2005
JOURNAL OF HAZARDOUS MATERIALS	20	32	1124	52	Zone 2	2016
ENVIRONMENTAL RESEARCH	17	31	994	39	Zone 2	2016
JOURNAL OF CHROMATOGRAPHY A	16	20	684	20	Zone 2	2006
ENVIRONMENTAL SCIENCE AND POLLUTION RESEARCH	13	23	564	40	Zone 2	2016
ECOTOXICOLOGY AND ENVIRONMENTAL SAFETY	13	24	601	36	Zone 2	2017
ENVIRONMENTAL SCIENCE & TECHNOLOGY LETTERS	12	15	666	15	Zone 2	2015
TOXICOLOGICAL SCIENCES	12	18	712	18	Zone 2	2003
MARINE POLLUTION BULLETIN	10	13	293	13	Zone 2	2019
NEUROTOXICOLOGY AND TERATOLOGY	8	9	482	9	Zone 3	2015
AQUATIC TOXICOLOGY	7	8	1020	8	Zone 3	2012
ARCHIVES OF ENVIRONMENTAL CONTAMINATION AND TOXICOLOGY	7	8	190	8	Zone 3	2017
JOURNAL OF APPLIED TOXICOLOGY	7	7	94	7	Zone 3	2016
WATER RESEARCH	7	10	596	10	Zone 3	2014

CHEMICAL ENGINEERING JOURNAL	6	6	359	6	Zone 3	2017
ENVIRONMENTAL HEALTH	6	7	223	7	Zone 3	2017
INTERNATIONAL JOURNAL OF ENVIRONMENTAL RESEARCH AND PUBLIC HEALTH	6	9	84	12	Zone 3	2018
ENVIRONMENTAL HEALTH PERSPECTIVES	5	7	423	7	Zone 3	2013
ENVIRONMENTAL MONITORING AND ASSESSMENT	5	5	103	5	Zone 3	2016
ENVIRONMENTAL TOXICOLOGY AND CHEMISTRY	5	9	223	9	Zone 3	2013
TALANTA	5	6	130	6	Zone 3	2016
TOXICOLOGY AND APPLIED PHARMACOLOGY	5	5	426	5	Zone 3	2006
ANALYTICAL AND BIOANALYTICAL CHEMISTRY	4	7	246	7	Zone 3	2011
CHEMICO-BIOLOGICAL INTERACTIONS	4	5	159	5	Zone 3	2008
INDOOR AIR	4	6	296	6	Zone 3	2012
INTERNATIONAL JOURNAL OF HYGIENE AND ENVIRONMENTAL HEALTH	4	9	116	9	Zone 3	2021
INTERNATIONAL JOURNAL OF MOLECULAR SCIENCES	4	7	162	7	Zone 3	2019
JOURNAL OF AGRICULTURAL AND FOOD CHEMISTRY	4	5	132	5	Zone 3	2018
JOURNAL OF ENVIRONMENTAL SCIENCES	4	5	56	5	Zone 3	2015
SCIENTIFIC REPORTS	4	6	172	6	Zone 3	2016
TOXICOLOGY LETTERS	4	6	235	6	Zone 3	2014
ACS APPLIED MATERIALS & INTERFACES	3	3	81	3	Zone 3	2011
CRITICAL REVIEWS IN ENVIRONMENTAL SCIENCE AND TECHNOLOGY	3	5	37	5	Zone 3	2022
ENVIRONMENTAL TOXICOLOGY	3	3	32	3	Zone 3	2017

JOURNAL OF ORGANIC CHEMISTRY	3	3	110	3	Zone 3	2004
LANGMUIR	3	3	137	3	Zone 3	2013
TOXICS	3	6	37	7	Zone 3	2021
ANALYTICA CHIMICA ACTA	2	3	78	3	Zone 3	2014
ANALYTICAL METHODS	2	2	19	2	Zone 3	2017
ARCHIVES OF TOXICOLOGY	2	4	79	4	Zone 3	2018
ATMOSPHERIC CHEMISTRY AND PHYSICS	2	2	27	2	Zone 3	2014
ATMOSPHERIC ENVIRONMENT	2	3	124	3	Zone 3	2007
BIOCHEMICAL PHARMACOLOGY	2	2	42	2	Zone 3	2014
BIOCHEMISTRY	2	2	7	2	Zone 3	2015
BULLETIN OF ENVIRONMENTAL CONTAMINATION AND TOXICOLOGY	2	2	93	2	Zone 3	2017
CHEMICAL RESEARCH IN TOXICOLOGY	2	3	45	3	Zone 3	2019

Table S8. Top 10 most highly cited literature related to OPFRs health risk research

Rank	Title	First author	Year	Journal/IF	Citation	DOI
1	Organophosphorus flame retardants and plasticizers: Sources, occurrence, toxicity and human exposure	Wei GL	2015	Environmental Pollution/IF=8.9	213	10.1016/j.envpol.2014.09.012
2	Organophosphate Ester Flame Retardants: Are They a Regrettable Substitution for Polybrominated Diphenyl Ethers?	Blum A	2019	Environmental Science &Technology Letters/IF=10.9	134	10.1021/acs.estlett.9b00582
3	Phosphorus flame retardants: properties, production, environmental occurrence, toxicity and analysis	Ike van der Veen	2012	Chemosphere/IF=8.8	124	10.1016/j.chemosphere.2012.03.067
4	Review of OPFRs in animals and humans: Absorption, bioaccumulation, metabolism, and internal exposure research	Hou R	2016	Chemosphere/IF=8.8	120	10.1016/j.chemosphere.2016.03.003
5	A review on organophosphate Ester (OPE) flame retardants and plasticizers in foodstuffs: Levels, distribution, human dietary exposure, and future directions	Li JH	2019	Environment International/IF=11.8	116	10.1016/j.envint.2019.03.009
6	Occurrence and Distribution of Organophosphate Flame Retardants/Plasticizers in Surface Waters, Tap Water, and Rainwater: Implications for Human Exposure	Kim UJ	2018	Environmental Science &Technology /IF=11.4	102	10.1021/acs.est.8b00727
7	Occurrence and distribution of organophosphate flame retardants (OPFRs) in soil and outdoor settled dust from a multi-waste recycling area in China	Wang Y	2018	Science of The Total Environment/IF=9.8	99	10.1016/j.scitotenv.2018.01.013
8	A review of organophosphate flame retardants and plasticizers in the environment: Analysis, occurrence and risk assessment	Wang X	2020	Science of The Total Environment/IF=9.8	98	10.1016/j.scitotenv.2020.139071
9	Organophosphate flame retardants (OPFRs): A review on analytical methods and occurrence in wastewater and aquatic environment	Pantelaki I	2019	Science of The Total Environment/IF=9.8	91	10.1016/j.scitotenv.2018.08.286
10	Exposure to organophosphate flame retardant chemicals in the U.S. general population: Data from the 2013-2014 National Health and Nutrition Examination Survey	Ospina M	2018	Environment International/IF=11.8	90	10.1016/j.envint.2017.10.001

Table S9. Top 100 most frequent keywords

Rank	Terms	Frequency	Rank	Terms	Frequency
1	organophosphate esters	327	51	ecological risk assessment	9
2	organophosphate flame retardants	241	52	indoor air	9
3	flame retardants	146	53	metabolomics	9
4	risk assessment	78	54	occupational exposure	9
5	human exposure	46	55	organic contaminants	9
6	triphenyl phosphate	41	56	pahs	9
7	endocrine disruption	40	57	tdcipp	9
8	indoor dust	33	58	air	8
9	zebrafish	24	59	alternative flame retardants	8
10	oxidative stress	23	60	drinking water	8
11	dust	21	61	e-waste	8
12	sediment	20	62	fish	8
13	metabolites	19	63	microplastics	8
14	bioaccumulation	18	64	ope	8
15	ecological risk	18	65	organophosphorus esters	8
16	exposure assessment	18	66	proteomics	8
17	health risk	18	67	sediments	8
18	plasticizers	18	68	biodegradation	7
19	soil	18	69	dna damage	7
20	toxicity	18	70	foodstuffs	7
21	developmental toxicity	17	71	gas-particle partitioning	7
22	exposure	17	72	gas chromatography	7
23	distribution	16	73	molecular docking	7
24	occurrence	16	74	pm2 5	7
25	spatial distribution	16	75	source	7
26	tdcpp	16	76	suspect screening	7
27	urine	16	77	triphenyl phosphate (tphp)	7
28	apoptosis	15	78	tris-(2-chloroisopropyl) phosphate	7
29	biomonitoring	15	79	tris(2-chloroethyl) phosphate	7
30	children	15	80	adsorption	6

31	pbdes	15	81	biomarkers	6
32	plasticizer	15	82	degradation	6
33	surface water	15	83	diphenyl phosphate	6
34	polybrominated diphenyl ethers	13	84	ecotoxicity	6
35	sources	13	85	endocrine disruptors	6
36	water	13	86	firefighters	6
37	metabolism	12	87	glucose homeostasis	6
38	neurodevelopment	12	88	nhanes	6
39	organophosphate	12	89	organic pollutants	6
40	organophosphorus flame retardant	12	90	organophosphate triesters	6
41	pregnancy	12	91	organophosphates	6
42	brominated flame retardants	11	92	particulate matter	6
43	cytotoxicity	11	93	pcbs	6
44	dietary exposure	11	94	pfas	6
45	house dust	11	95	phthalate esters	6
46	tcep	11	96	pregnant women	6
47	urinary metabolites	11	97	rice	6
48	emerging contaminants	10	98	risk	6
49	organophosphorus flame retardants (opfrs)	10	99	seasonal variation	6
50	behavior	9	100	sludge	6

Table S10. Main clustering and internal keywords

Cluster ID	Label	Size	Average year	Main keywords
0	Health risk assessment	98	2012	organophosphate ester, risk assessment, toxicity, water, air, dust, spatial distribution, seasonal variation, metabolism, bioaccumulation, developmental neurotoxicity
1	Human exposure	82	2014	polybrominated diphenyl ether, indoor dust, halogenated flame retardant, microenvironment, serum, chromatography mass spectrometry, sewage treatment plant, particulate matter, association
2	Endocrine disruption	70	2013	triphenyl phosphate, urinary metabolite, oxidative stress, expression, pathway, endocrine disrupting chemical, prenatal exposure, zebrafish, in vitro metabolism
3	mRNA expression	51	2011	Fate, growth, thyroid hormone, mice, nuclear receptor, chicken embryo, transcription factor, lipid metabolism
4	Neuropathy target esterase	34	2010	Mechanism, brain, protein, neurotoxic esterase, butyrylcholinesterase, acetylcholinesterase knockout mouse, delayed neuropathy, diabetic neuropathy
5	Acetylcholinesterase	34	2004	Analog，acid amide hydrolase, amino acid substitution, anhydride hydrolase activity, enzymatic hydrolysis, cannabinoid receptor
6	quantitative structure-activity relationship (QSAR)	29	2014	multi residue analysis, soil, probe, extraction, cell, partial least squares, binding affinity, free energy relationship, bioavailability
7	Mass spectrometry	25	2014	south china, persistent organic pollutant, dietary intake, blood, pressurized liquid extraction, biomarker, gas chromatographic mass spectrometry, human hair and nail
8	Orgnophosphate ester metabolites	25	2013	Phosphate, rat, neurotoxicity, hormone level, acetylcholinesterase, temporal variability, pregnant women, gene transcriptional analysis, adolescence
9	In vitro	25	2013	Gene, basic science, chondrocyte death, cancer, thyroid follicle, hormone receptor, diphenyl ether
10	Sample preparation	22	2012	liquid chromatography, tandem mass spectrometry, flame retardant metabolite, emerging contaminant, analytical method, human matrice, tissue disposition

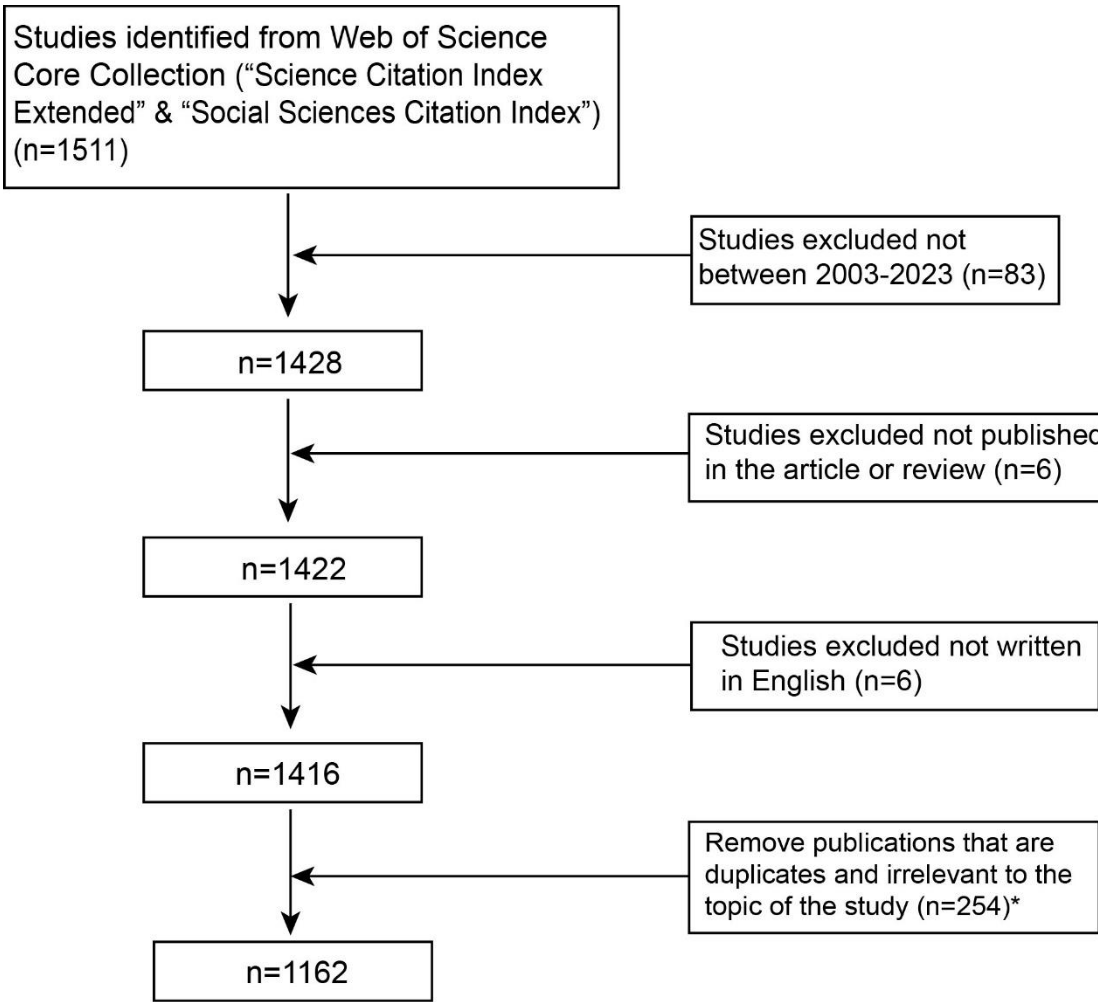

Figure S1. Publication selection and flow chart of the research framework. *: In general, articles that dealt only with the environmental distribution, environmental behavior, physical and chemical properties, degradation mechanisms, and purely analytical methods or model development of organophosphate flame retardants were judged to be irrelevant to the purpose of this study and were excluded from the analysis.

(A)

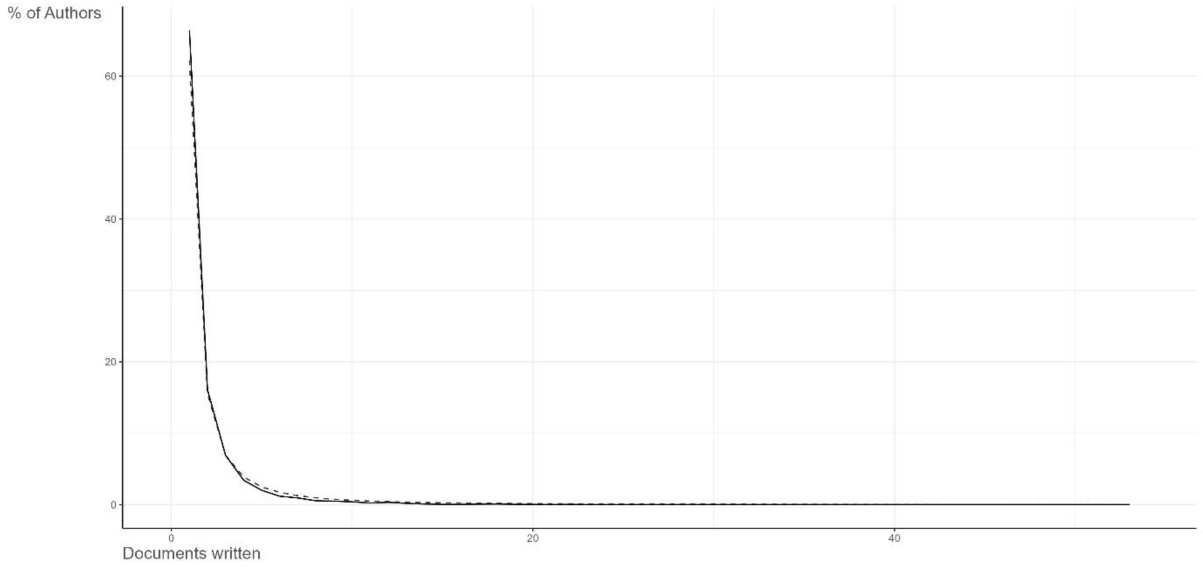

(B)

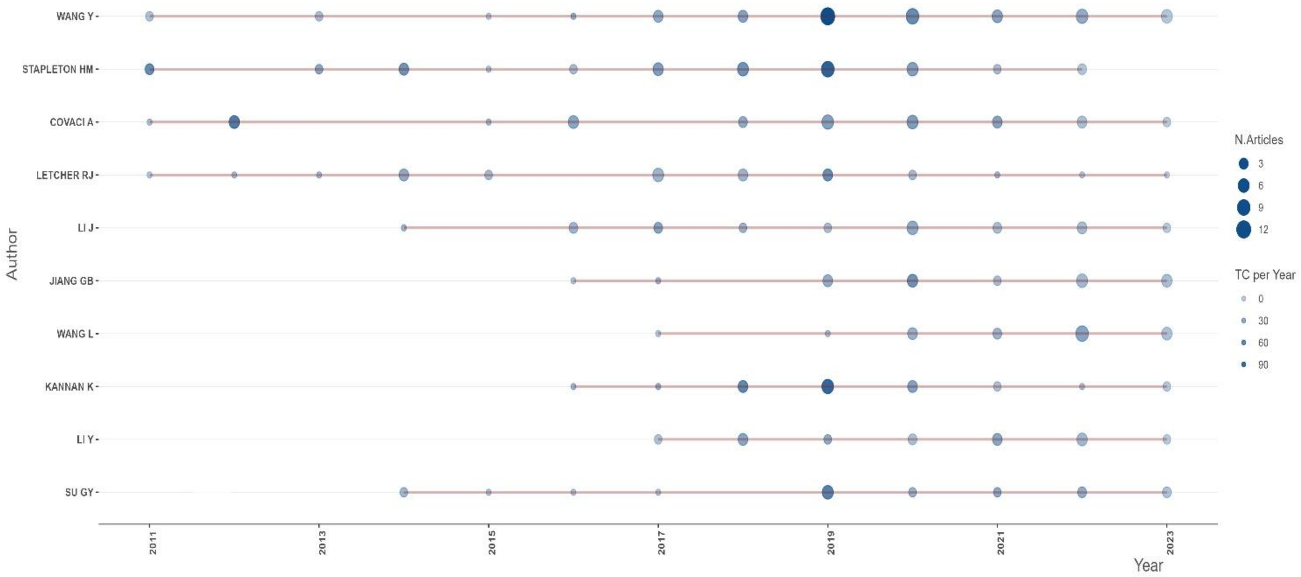

Figure S2. Author analysis. (A) Lotka's law fitting curve; (B) Top 10 author's production over time; note: the size of the circle represents the number of articles published in the year, and the shade of the color represents the average number of citations per year.

(A)

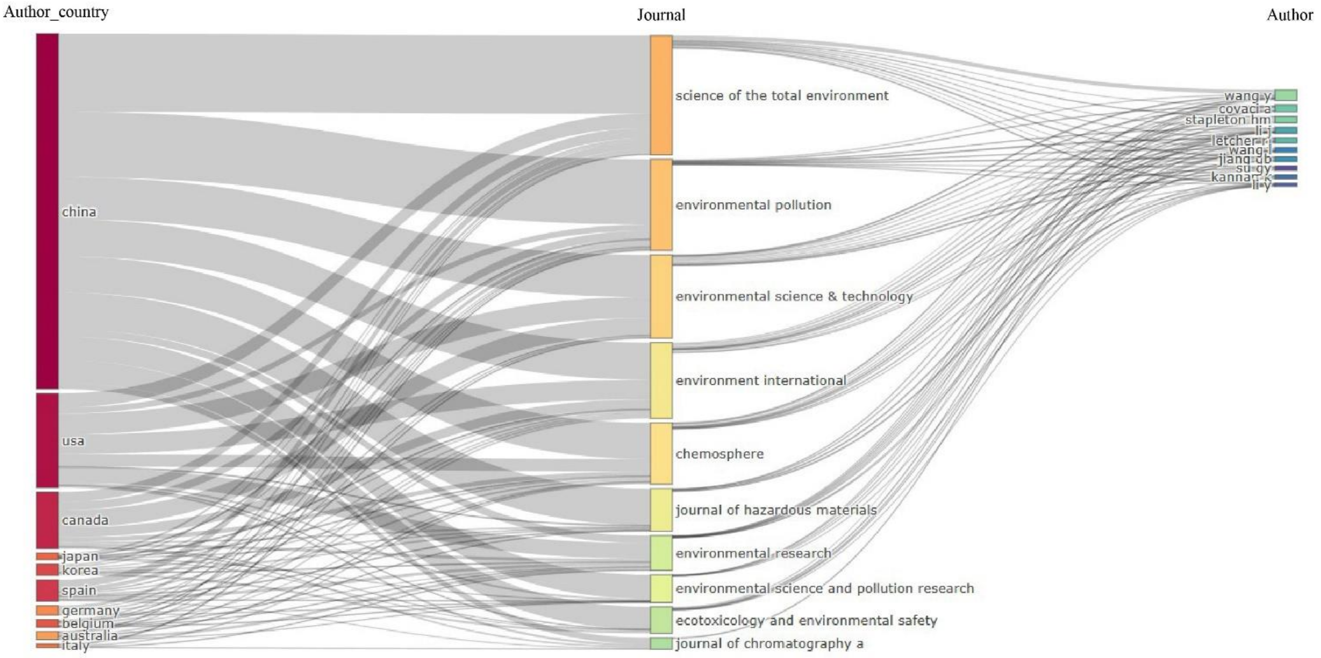
Author_country
Journal
Author
china
usa
canada
japan
korea
spain
germany
belgium
australia
italy
science of the total environment
environmental pollution
environmental science & technology
environment international
chemosphere
journal of hazardous materials
environmental research
environmental science and pollution research
ecotoxicology and environmental safety
journal of chromatography a
wang y
covaci a
stapleton hm
li j
letcher r
wang l
jiang gb
su gy
kannan k
li y

(B)

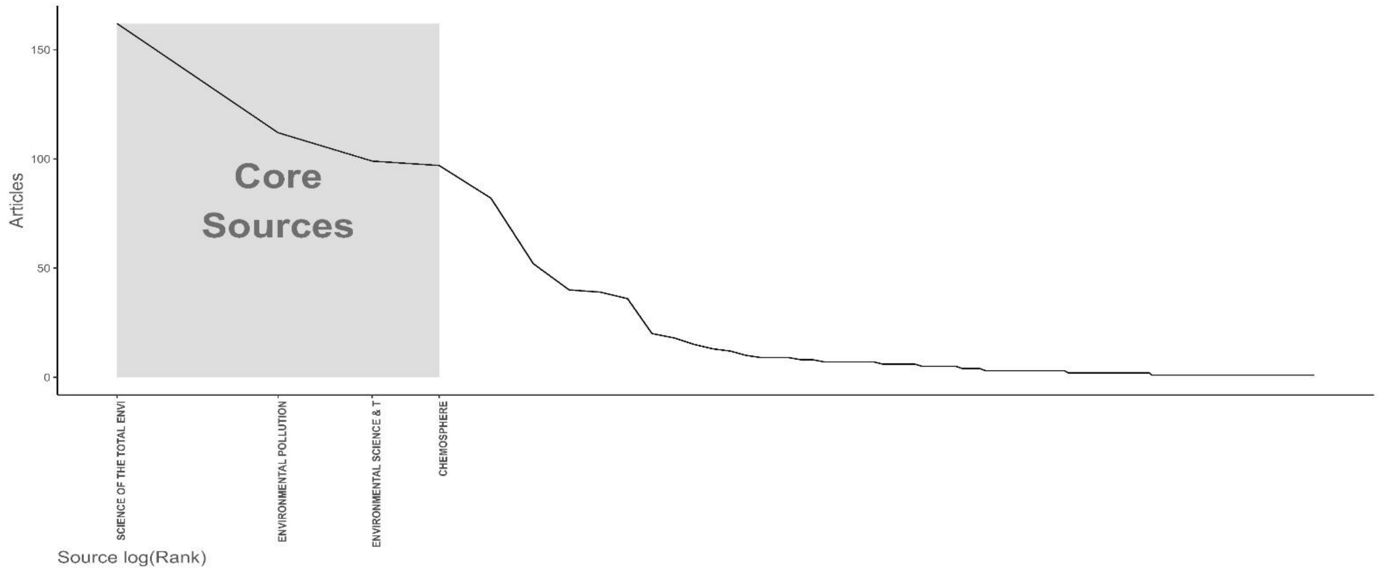
Articles
150
100
50
0
Core Sources
SCIENCE OF THE TOTAL ENVI
ENVIRONMENTAL POLLUTION
ENVIRONMENTAL SCIENCE & T
CHEMOSPHERE
Source log(Rank)

(C)

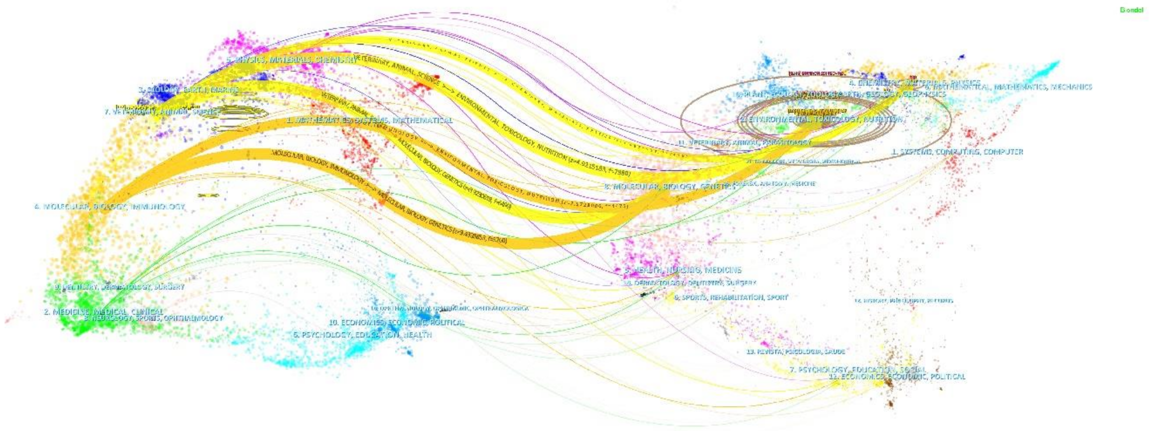

Figure S3. Source analysis. (A) Country-Journal-Author Sankey diagram;Note: The length of a rectangular node is proportional to the frequency of occurrence, and the width of the line between nodes is proportional to the number of connections; (B) Bradford's law analysis based on the R-bibliometrix package. Gray represents the core area of the journal; (C) Discipline-journal dual-mapping overlay analysis. Different colors on the left side represent different types of citing journals, and different colors on the right side represent different types of cited journals.

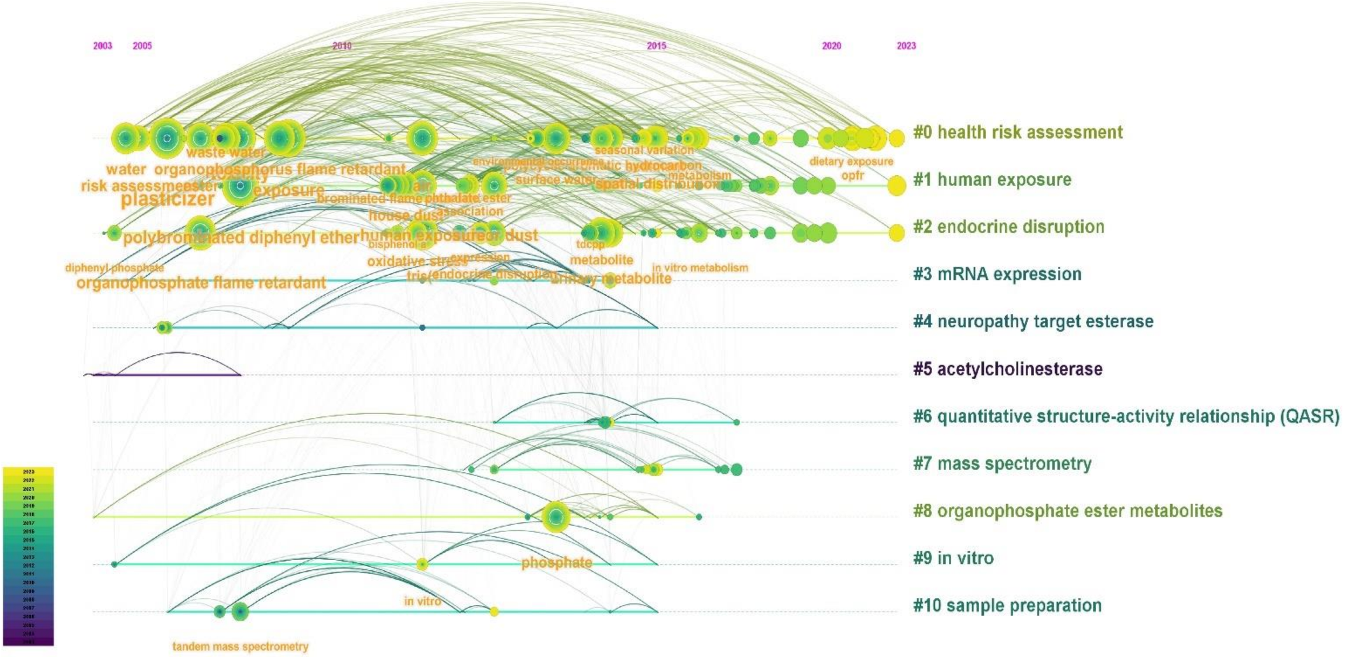

Figure S4. Keyword clustering - timeline diagram.

Reference

1. Zeng, N.; Sun, J.-X.; Liu, C.-Q.; Xu, J.-Z.; An, Y.; Xu, M.-Y.; Zhang, S.-H.; Zhong, X.-Y.; Ma, S.-Y.; He, H.-D.; Wang, S.-G.; Xia, Q. D. Knowledge Mapping of Application of Image-Guided Surgery in Prostate Cancer: A Bibliometric Analysis (2013-2023). *Int. J. Surg. Lond. Engl.* **2024**. https://doi.org/10.1097/JS9.0000000000001232.
2. Hirsch, J. E. An Index to Quantify an Individual's Scientific Research Output. *Proc. Natl. Acad. Sci. U. S. A.* **2005**, *102* (46), 16569–16572. https://doi.org/10.1073/pnas.0507655102.
3. Egghe, L. Theory and Practise of the *g*-Index. *SCIENTOMETRICS* **2006**, *69* (1), 131–152. https://doi.org/10.1007/s11192-006-0144-7.
4. Newman, M. E. J. Modularity and Community Structure in Networks. *Proc. Natl. Acad. Sci.* **2006**, *103* (23), 8577–8582. https://doi.org/10.1073/pnas.0601602103.
5. Chen, C.; Song, M. Visualizing a Field of Research: A Methodology of Systematic Scientometric Reviews. *PloS One* **2019**, *14* (10), e0223994. https://doi.org/10.1371/journal.pone.0223994.
